# ﻿A review of the *semipunctata* species group within the genus *Lilioceris* Reitter, 1913 (Coleoptera, Chrysomelidae)

**DOI:** 10.3897/zookeys.1195.114392

**Published:** 2024-03-20

**Authors:** Yuan Xu, Gexia Qiao, Hongbin Liang

**Affiliations:** 1 Key Laboratory of Zoological Systematics and Evolution, Institute of Zoology, Chinese Academy of Sciences, Beijing 100101, China Institute of Zoology, Chinese Academy of Sciences Beijing China; 2 College of Life Science, University of Chinese Academy of Sciences, Beijing 100049, China University of Chinese Academy of Sciences Beijing China

**Keywords:** Criocerinae, key, new record, new synonym

## Abstract

A new species group of *Lilioceris* Reitter, 1913 is proposed and reviewed, the *semipunctata* group. It includes eleven species: *L.atrilateralis* Kimoto & Takizawa, 1973, *L.consentanea* (Lacordaire, 1845), *L.dentifemoralis* Long, 1988, *L.discrepens* (Baly, 1879), *L.jianfenglingensis* Long, 1988, *L.latissima* (Pic, 1932), *L.lianzhouensis* Long, 2000a, *L.rondoni* Kimoto & Gressitt, 1979, *L.rufometallica* (Pic, 1923), *L.semipunctata* (Fabricius, 1801), and *L.yuae* Long, 2000b. *Liliocerisdiscrepens* and *L.rondoni* were new records from China. Two synonyms are proposed: *Liliocerisxinglongensis* Long, 1988, **syn. nov.** of *L.consentanea* (Lacordaire, 1845), and *Criocerisrufimembris* Pic, 1921, **syn. nov.** of *L.semipunctata*. An identification key, descriptions, habitus photographs, geographic distributions, host plants and habitats (if available), are provided for these species.

## ﻿Introduction

*Lilioceris* Reitter, 1913 has a high number of species in Criocerinae Latreille, 1804, which to date contains approximately 150 species worldwide, and at least 70 species in Asia (Monrós 1960; Heinze and Pinsdorf 1962; Gressitt 1965; Warchałowski 2011; Bezděk and Schmitt 2017). The genus is widely distributed in tropical and subtropical parts of the world, with the highest species diversity found in the Oriental Region. There are many taxonomic works focusing on regional species of the genus (e.g., Jacoby 1904, 1908; Gressitt and Kimoto 1961; Heinze and Pinsdorf 1962, 1963, 1964; Kimoto and Gressitt 1979; Tishechkin et al. 2011; Warchałowski 2011; Xu et al. 2021), but still many similar species are difficult to identify based on existing keys, and more revisionary work on species group is needed.

At present, three species groups in *Lilioceris* have been recognized and reviewed: the *L.impressa* group (Tishechkin et al. 2011), the *L.neptis* group (Xu et al. 2021), and the *L.sinica* group (Xu and Liang 2022). Here, we established the fourth, the *semipunctata* group, including eleven species. Some species have long been confused and misused, and the characteristics listed by different researchers are different. For example, Kimoto and Gressitt (1979) listed simple characteristics of *L.discrepens* and *L.latissima* in a key, and provided illustrations for the sternum and episternum. However, we examined and compared the types (Figs 9, 10) and found that their identifications were wrong. Therefore, these species need to be revised.

In this article, we compare types and re-identify syntypes, establish a new species group, propose two synonyms, and provide a key to aid identification.

## ﻿Materials and methods

Specimens from several museums and collections were examined. The collections cited in this article are indicated by the following abbreviations:

**BSM**Bishop Museum, Honolulu, Hawaii, USA;

**IZCAS**National Zoological Museum, Institute of Zoology, Chinese Academy of Sciences, Beijing, China;

**MBSU** Museum of Biology, Sun Yat–Sen University, Guangzhou, China;

**MHU** Museum of Hebei University, Baoding, China;

**MNHN**Museum National d’Histoire Naturelle, Paris, France;

**NHML**Natural History Museum, London, UK;

**ZMUK** Zoological Museum of Kiel University, Kiel, Germany;

**SEHU**Hokkaido University, Sapporo, Japan.

Except as noted, all specimens examined are deposited in IZCAS.

Dry specimens were soaked in hot water for 1–2 h to soften the body. The abdomen was opened at its latero-apical margin and genitalia were pulled out using forceps. Genitalia were soaked in warm 10% KOH for 1 h, and dyed in Chlorazol Black E. The basal orifice of the median lobe was injected with 100% ethanol with a micro-injector until the internal sac was fully everted. The median lobe with its everted internal sac was photographed using a large depth-of-field 3D digital microscope (Keyence VHX–1000C), and finally edited in Photoshop^©^. A microvial with genitalia was pinned to the specimen from which the genitalia were removed for storage.

Body length (**BL**) was measured from the anterior margin of the labrum to the apex of the elytra; body width (**BW**) was measured along the greatest elytral width. Other methods of specimen observation and preparation follow previous publications (Tishechkin et al. 2011; Li et al. 2013). Morphological terminology follows Chou et al. (1993), Tishechkin et al. (2011) and Schmitt and Uhl (2015).

## ﻿Results

**The *Liliocerissemipunctata* species group of *Lilioceris* Reitter, 1913**

**Diagnosis.** Species of this group share the following characters: 1) body almost brownish red, some with metallic luster, some of the lateral metasternum or base of abdominal sternites dark; 2) antennae more or less flattened; 3) pronotum without distinct transverse impression; 4) scutellum densely pubescent; 5) elytral punctures diminishing posteriorly and absent at 1/2, 1/3, or 1/4 of apex; 6) apical portion of mesosternal process narrow, obliquely pointed, not horizontally connected with metasternum; 7) medium to large size, body length more than 7.5 mm.

**Host plants.** The known host plants of this species group are *Smilax* spp. (Smilacaceae), except for *L.consentanea*, whose host plant is *Cycasrevoluta* Thunb. (Cycadaceae).

**Remarks.** This group is distinctly different from the *impressa* group in having elytral punctures absent at ~ 1/3–1/4 of apex; scutellum densely pubescent (in the *impressa* group, the elytral punctures distinct at apex; scutellum nearly glabrous). It is different from the *neptis* group in having apical portion of the mesosternal process narrow, obliquely pointed, and not horizontally connected with metasternum (in the *neptis* group, the apical portion of mesosternal process is strongly widened and convex, horizontally connected with the metasternum). It is different from the *sinica* group in having elytral punctures absent at 1/3–1/4 of apex; antennomeres 5–10 flat (in the *sinica* group, the elytral punctures distinct at apex; antennomers 5–10 cylindrical).

### ﻿Key to species of the *Liliocerissemipunctata* species group

**Table:** 

1	Femora of mid- and hind legs with a ventral tooth (Fig. 28C)	***L.dentifemoralis***
–	Femora of mid- and hind legs without tooth (Figs 27C, 29–36C)	**2**
2	Anterior angles of pronotum more protruding, rounded (Fig. 37B)	***L.consentanea***
–	Anterior angles of pronotum less protruding, angulate (Figs 38–46B)	**3**
3	Metasternum glabrous (Fig. 41C)	***L.latissima***
–	Metasternum pubescent (Figs 37–40C, 42–46C)	**4**
4	Lateral transverse impressions on abdominal sternites 1–4 distinct, glabrous and large, area outside the transverse impression pubescent (Figs 29, 30A, 32A, 34A)	**5**
–	Lateral transverse impressions on abdominal sternites 1–4 indistinct or absent, sternites laterally entirely pubsecent (Figs 33A, 35, 36A)	**8**
5	Antennomeres 5–10 ~ 1.0–1.2× as long as wide (Figs 29, 30D, 34D)	**6**
–	Antennomeres 5–10 ~ 1.8× as long as wide (Fig. 32D)	***L.lianzhouensis***
6	Body length > 10.0 mm; pronotal disc with four or five rows of dense punctures (Fig. 39B)	***L.discrepens***
–	Body length < 10.0 mm; pronotal disc with two rows of sparse punctures (Figs 40B, 44B)	**7**
7	Body with strong cupreous metallic luster; punctures of elytra diminishing posteriorly, absent on apical 1/4 (Fig. 34B)	***L.rufometallica***
–	Body brown, at most with weak blue metallic luster; punctures of elytra diminishing posteriorly, absent on apical 1/2–1/3 (Fig. 30B)	***L.jianfenglingensis***
8	Vertex strongly raised (Fig. 43A)	***L.rondoni***
–	Vertex slightly raised or flat (Figs 45, 46A)	**9**
9	Antennae, head, pronotum, leg and lateral metasternum with weak blue metallic luster (Figs 25, 26); lateroposterior corner of metasternum with a short oblique strip of pubescence (Fig. 46C)	***L.yuae***
–	Body without metallic luster, base of abdominal sternites dark (Figs 21, 22), lateral metasternum nearly black; metepisternum with a long narrow strip of pubescence (Fig. 45C)	**10**
10	Femora unicolor, brownish red	***L.semipunctata***
–	Femora bicolor, black with middle brownish red in ventral	***L.atrilateralis***

### ﻿Taxonomic account

#### ﻿ Lilioceris atrilateralis

Taxon classificationAnimaliaColeopteraChrysomelidae

Kimoto & Takizawa, 1973

1F4937B2-6DE9-5BC6-A99B-7CD37EFFA7CA

Figs 1 , 2

Lilioceris atrilateralis Kimoto & Takizawa, 1973: 171 (Nepal).

##### Type material examined.

***Holotype*** of *Liliocerisatrilateralis* (SEHU, photo), Nepal, T. Kumata / Balaju Kathmandy, 1968.IV.16 / 5 / *Liliocerisatrilateralis* Kimoto & Takizawa, 1973 / Holotype / Holotype, appended label by ÔHARA, INAPI, KANBE AUZUKI and HIRONACA, 2007 / 0000001233 Sys. Ent, Hokkaido Univ. Japan [SEHU].

**Figures 1, 2.**
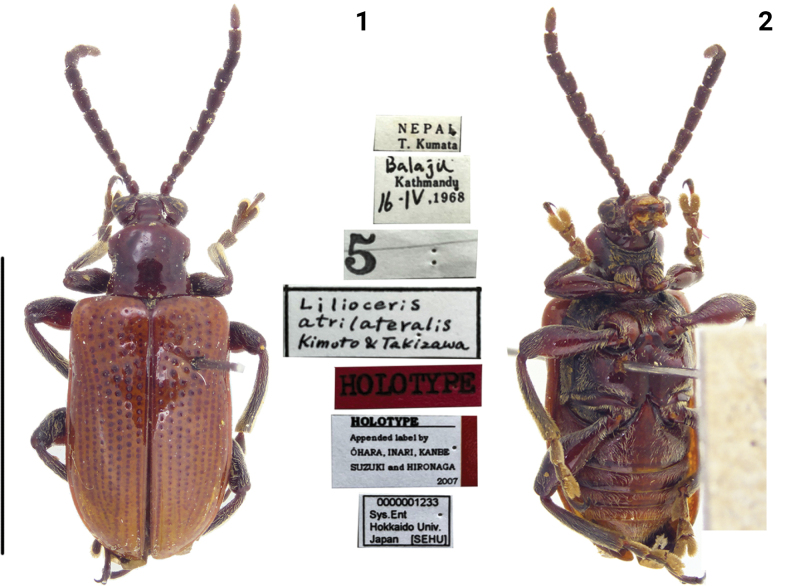
Habitus of *Liliocerisatrilateralis*, holotype, Nepal (Kathmandy). Photographed by Takuya Takemoto (SEHU). Scale bar: 5.0 mm.

##### Remarks.

This species is very similar to *L.semipunctata* according to the original description and photographs of the holotype, but differs by the black femora with its middle brownish red in ventral view; sides of the abdomen are black. In *L.semipunctata*, the femora are brownish red; sides of the abdomen are brownish red. For this species, we have not collected sufficient specimens; therefore, the species is only listed and not treated in detail.

##### Distribution

**(Fig. 68).** Nepal (Kimoto and Takizawa 1973).

#### ﻿ Lilioceris consentanea

Taxon classificationAnimaliaColeopteraChrysomelidae

(Lacordaire, 1845)

222F7358-276C-5E22-8D94-EECE86F40561

Figs 3–6 , 27A–D , 37A–C , 56A–D , 58A–C , 67 , 69A, B

Crioceris consentanea Lacordaire, 1845: 561 (Vietnam).
Lilioceris consentanea : Kimoto and Gressitt 1979: 222.
Lilioceris xinglongensis Long, 1988: 232 (China: Hainan, holotype, male), syn. nov.

##### Type material examined.

***Syntype*** of *Liliocerisconsentanea* (NHML, photo), Type / Coll E. Chev. t / Criocerisconsentanea Lac. Cochinchine ex mus Guerini, Type 20 / Criocerisconsentanea Lac. Type / BMNH(E)1344910 / 37; ***holotype*** of *Liliocerisxinglongensis* (MBSU), Holotype, *Liliocerisxinglongensis* Long, ♀, Jianguo Long det. / Hainan, Xinglong, 1980.IX.29–X.3. Shiyang Xia coll. / 603 / En–207209 SYS.

**Figures 3–6.**
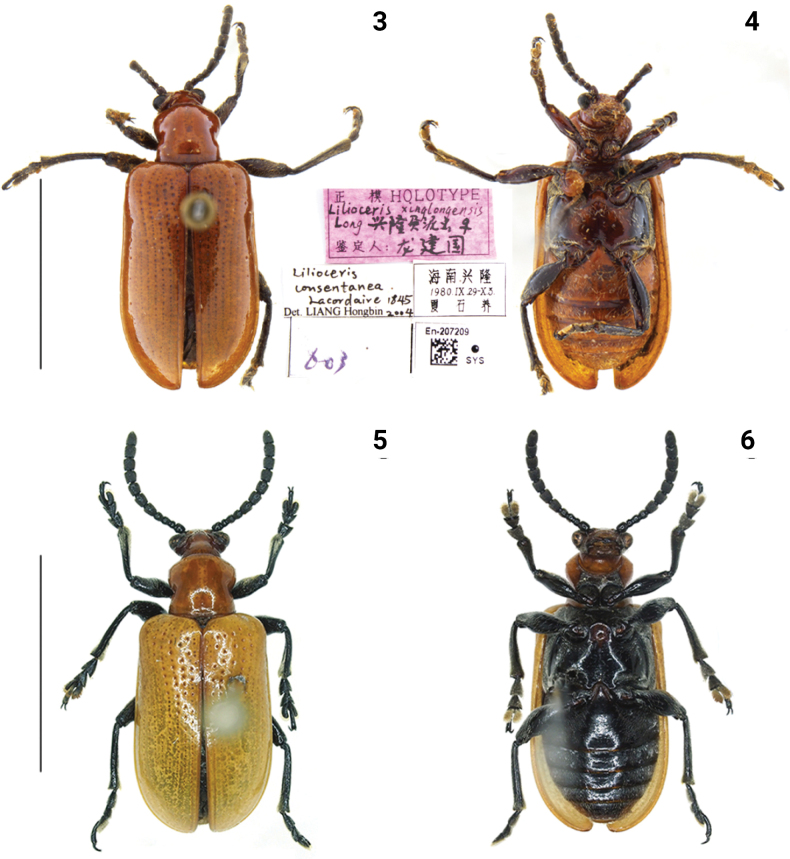
Habitus of *Lilioceris* spp. **3, 4***L.xinglongensis*, holotype, China (Hainan) **5, 6***L.consentanea*, specimen from China (Yunnan). Photographed by Yuan Xu. Scale bars: 5.0 mm.

##### Other material examined.

Total 152 specimens. **China**: **Hainan**: 12♀16♂, Xinglong Farm / 1974.IV, host plant: *Cycasrevoluta*; 14♀27♂, Diaoluo Shan Forestry Bureau, 18.66590°N, 109.93044°E / 85 m, 2007.XII.10, Zhu XY and Yang GY coll., Inst. of Zoology, CAS; 1♀, Lingshui, Diaoluo Shan, 920 m, 2007.V.3, Li Xian / IOZ(E)1880967; 1♂, Lingshui, Diaoluo Shan, 920 m, 2007.V.3, Li Xian / IOZ(E)1880965; 1♂, Lingshui, Diaoluo Shan, 920 m, 2007.V.3, Li Xian / IOZ(E)1880968; 1♀, Lingshui, Diaoluo Shan, 920 m, 2007.V.3, Li Xian / IOZ(E)1880969; 1♀, Lingshui, Diaoluo Shan, 920 m, 2007.V.3, Li Xian / IOZ(E)1880970; 1♂, Lingshui, Diaoluo Shan, 920 m, 2007.V.3, Li Xian / IOZ(E)1880971; 1♂, Lingshui, Diaoluo Shan, 920 m, 2007.V.3, Li Xian / IOZ(E)1880972; 1♂, Lingshui, Diaoluo Shan, 920 m, 2007.V.3, Li Xian / IOZ(E)18809732; 1♂, Lingshui, Diaoluo Shan, 920 m, 2007.V.3, Li Xian / IOZ(E)1880974; 1♂, Lingshui, Diaoluo Shan, 920 m, 2007.V.3, Li Xian / IOZ(E)1880975; 1♂, Lingshui, Diaoluo Shan, 920 m, 2007.V.3, Li Xian / IOZ(E)1880976; 1♂, Lingshui, Diaoluo Shan, 920 m, 2007.V.3, Li Xian / IOZ(E)1880977; 1♂, Lingshui, Diaoluo Shan, 920 m, 2007.V.3, Li Xian / IOZ(E)1880978; 1♀, Lingshui, Diaoluo Shan, 920 m, 2007.V.3, Li Xian / IOZ(E)1880979; 1♀, Lingshui, Diaoluo Shan, 920 m, 2007.V.3, Li Xian / IOZ(E)1880980; 1♀, Lingshui, Diaoluo Shan, 920 m, 2007.V.3, Li Xian / IOZ(E)1880981; 1♂, Lingshui, Diaoluo Shan, 920 m, 2007.V.3, Li Xian / IOZ(E)1880982; 1♀, Lingshui, Diaoluo Shan, 920 m, 2007.V.3, Li Xian / IOZ(E)1880983; 1♂, Lingshui, Diaoluo Shan, 920 m, 2007.V.3, Li Xian / IOZ(E)1880984; 1♂, Lingshui, Diaoluo Shan, 920 m, 2007.V.3, Li Xian / IOZ(E)1881004; 2♂, Lingshui, Diaoluo Shan, 921 m, 2007.V.3, Liu Y. and Shi Hongliang coll., Inst. of Zoology, CAS; 2♂, Lingshui, Diaoluo Shan, 921 m, 2007.V.3, Liu Y. and Shi Hongliang coll., Inst. of Zoology, CAS; 2♀, Lingshui, Diaoluo Shan, 921 m, 2007.V.3, Liu Y. and Shi Hongliang coll., Inst. of Zoology, CAS; 15♀26♂, Lingshui, Mt. Xin–an, on vegetation, 18.72510°N, 109.86861°E / 921 m, 2007.03.25, Day, Shi H.L., Yuan F. coll., Institute of Zoology, Chinese Acad. Sciences; 4♀5♂, Lingshui, Diaoluo Shan, Xin–an, on vegetation, 18.72510°N, 109.86861°E / 921 m, 2007.III.25, Hongliang Shi and Feng Yuan coll.; 3♀5♂, Lingshui, Diaoluo Shan, 18.72467°N, 109.86804°E, 920 m, Rui Chen and Ye Liu coll.; **Yunnan**: 1♀, Xishuangbanna, Menglun, 600 m / 1994.IV.24, Huanli Xu coll. / Liliocerisconsentanea, det. Liang H.B.; 1♀, Xishuangbanna, Menghun, 750 m / 1958.VI.5, Xuwu Meng coll.; 1♂, Xishuangbanna, Xiaomengyang, 850 m / 1957.X.13, Lingchao Zang coll.; 1♂, Menghai, 1060 m, 1980.V.1 / Liliocerisconsentanea, det. Peiyu Yu; 1♂, Xishuangbanna, Menghai, 1200–1600 m, 1958.VII.25, Fuji Pu coll. / Liliocerisconsentanea, det. Peiyu Yu; 1♂, Xishuangbanna, Xiaomengyang, 850 m / 1957.X.26, Lingchao Zang coll; **Laos**: 1 specimen (NHML), Luang Prabang. Sept. 1917. R.V. de Salvaza. / Criocerisconsentanea Lac. / 1344970, BMNH(E).

##### Diagnosis.

Anterior angles of pronotum rounded, pronotal disc with two rows of fine punctures. Humeral groove of elytra indistinct, punctures sparse and diminishing posteriorly, absent on apical 1/3. Femora of mid- and hind legs without tooth. Lateral metasternum with a wide strip of pubescence.

##### Redescription.

BL = 8.5–9.2 mm, BW = 3.5–4.0 mm. ***Body*** mostly brownish red, antennae, legs, mesoepisternum, mesoepimeron, and lateral metaepisternum black; in some specimens, ventral surface black.

***Head*** (Figs 3, 5, 37A). Vertex flat, with a shallow groove in the middle, sparsely punctate and pubescent laterally; frontoclypeal area triangular, lateral disc with punctures and setae; labrum transverse, with sparse setae; antennae nearly 1/3 length of body, antennomeres 1–4 nearly globular, antennomere 2 shortest, antennomeres 5–10 slightly longer than wide.

***Pronotum*** (Figs 3, 5, 37B). Anterior angles distinctly rounded, not protruding, posterior angles slightly protruding; sides strongly constricted in the middle; disc slightly raised; middle of disc with two rows fine punctures. Scutellum triangular and pubescent.

***Elytra*** (Figs 3, 5, 27B). Humeri protruding, humeral groove and basal impression indistinct; strial punctures sparse and large in the base, diminishing posteriorly, absent on apical 1/3; intervals smooth; epipleura raised, with a row of fine punctures.

***Mesosternum pubescent*.** Lateral metasternum with a long strip of pubescence, extending from the posterior to anterior margin. Metepisternum densely pubescent (Fig. 37C).

***Abdominal sternite*** (Fig. 27A). Densely pubescent, lateral transverse impressions absent.

***Leg*** (Fig. 27C). Femora with dense pubescence on the dorsal surface, with sparse pubescence on the ventral surface, without tooth.

***Male genitalia*** (Fig. 56A–D). Ostium occupying 1/4 length of median lobe (Fig. 56A); apex hooked (Fig. 56B); tegmen Y-shaped, basal piece of tegmen oval and broad, lateral lobes distinctly sclerotized and combined with second connecting membrane; internal sac membranous, with distinct dorsal and ventral sclerites, posterior part of dorsal sclerite in dorsal view parallel, ventral sclerite strongly extended and tubular (= flagellum), median sclerite very small (Fig. 56C, D).

***Female reproductive organs*** (Fig. 58A–C). Tergites 8 and 9, sternites 8 and 9 sclerotized, posterior areas of tergite 8, sternite 8, and apodemes with pubescence, spiculum gastrale Y-shaped and strong; ovipositor with sparse pubescence, distal part of ovipositor cylindrical, short and with a protuberance; spermatheca simple and curved.

##### Distribution

**(Fig. 67).** China (Yunnan, Hainan, Fujian (Bezděk and Schmitt 2017)); Laos; Vietnam (Warchałowski 2011).

##### Host plant and habitat

**(Fig. 69A, B).** This species fed on *Cycasrevoluta* Thunb. according to observation of HBL in Diaoluo Shan (Hainan). Diaoluo is one of the most well-preserved areas of tropical rainforest in China, located in the southeast of Hainan. The area has an oceanic tropical monsoon climate with abundant rainfall. The habitat of this species is open, composed of tall trees, woody vines, and weeds. It also occurred on *C.revoluta* planted in the garden.

##### Remarks.

This species can be easily recognized by its anterior angles of the pronotum being particularly rounded (Fig. 37B), and its male genitalia with three sclerites that are not clearly separated. The male genitalia are similar to that of *L.yuae* Long, 2000b, but differ by the dorsal sclerite being cruciform in dorsal view (Fig. 56C). In *L.yuae*, the dorsal sclerite is cuneiform in dorsal view (Fig. 55C).

The body color of this species is variable. The abdomen and legs of the specimens from Hainan are brownish red, while the specimens from Yunnan are completely black. We have compared the genitalia of specimens from Hainan and Yunnan and found no differences. We compared the type of *Liliocerisxinglongensis* Long, 1988 (Figs 3, 4) with that of *L.consentanea*, and found no significant differences; the two species are therefore treated as conspecific.

#### ﻿ Lilioceris dentifemoralis

Taxon classificationAnimaliaColeopteraChrysomelidae

Long, 1988

9540D7E6-561D-5039-87DA-425ABA9D8B70

**Figures 7–10.**
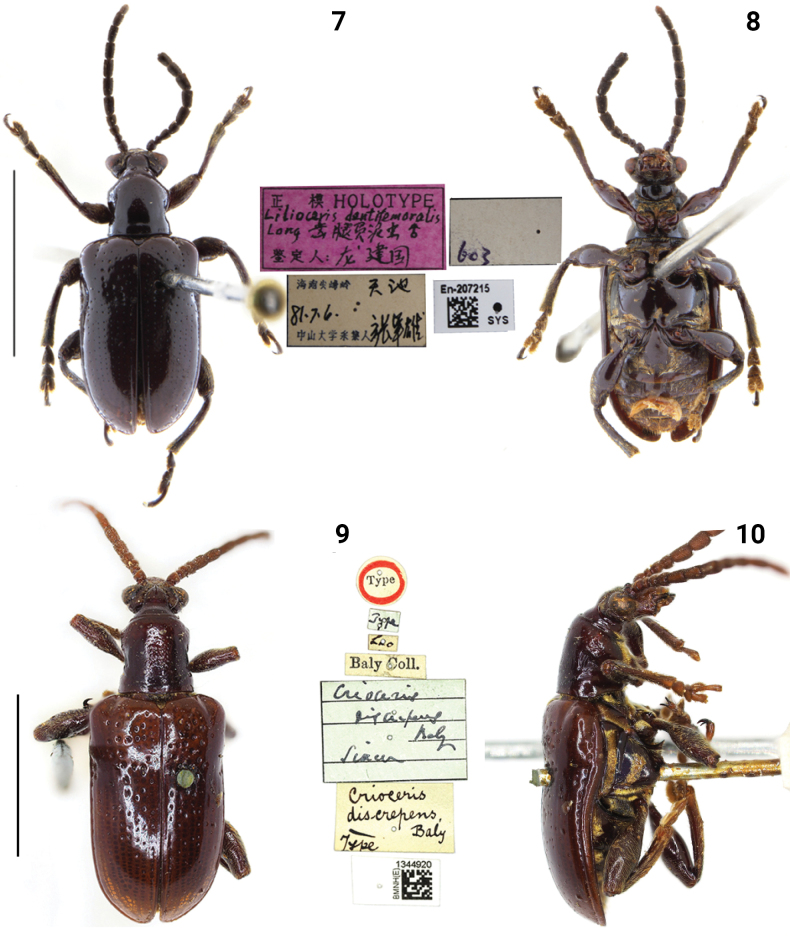
Habitus of *Lilioceris* spp. **7, 8***L.dentifemoralis*, holotype, China (Hainan), photographed by Yuan Xu **9, 10***L.discrepens*, syntype, Laos, photographed by Hongbin Liang. Scale bars: 5.0 mm.

Figs 7 , 8 , 28A–D , 38A–C , 47A–D , 57A–C , 68

Lilioceris dentifemoralis Long, 1988: 231 (China: Hainan).

##### Type material examined.

***Holotype*** of *Liliocerisdentifemoralis* (MBSU, photo), Hainan, Jianfengling, Tianchi, 1981.7.6, Junxiong Zhang coll. / holotype, Liliocerisdentifemoralis Long ♂, Jianguo Long det. / 603 / En–207215 SYS; ***allotype*** of *Liliocerisdentifemoralis* (MBSU, photo), Hainan, Jianfengling, 1964.V.3–5, Hui Ren coll. / Allotype, Liliocerisdentifemoralis Long ♀, Jianguo Long det. / En–207214 SYS.

##### Other material examined.

Total 6 specimens. **China**: 1♀, Hainan, Jianfengling, 1981.8.13 / Maobin Gu collector / Liliocerisdentifemoralis det. Liang H.B., 2020; 1♀, Hainan, Jianfengling, 1964.V.10 / Tailu Chen collector/ Liliocerisdentifemoralis det. Peiyu Yu/ Liliocerisdentifemoralis, compared with type, det. Liang H.B., 2004.3; **Vietnam**: 1♀, Tonkin, Cho Ganh, L Duport / Liliocerisdentifemoralis, det. Liang H.B.; 1♀2♂, Tonkin.

##### Diagnosis.

Anterior angles of pronotum slightly protruding, pronotal disc with two or three irregular rows fine punctures. Humeral groove of elytra distinct, punctures of elytra diminishing posteriorly, absent on apical 1/4. Femora of mid- and hind legs with tooth. Lateral metasternum with a wide strip of pubescence.

##### Redescription.

BL = 7.0–8.0 mm, BW = 3.5–4.0 mm. ***Body*** brownish red.

***Head*** (Figs 7, 38A). Vertex flat, with a shallow groove in the middle, sparse punctate and pubescent laterally; frontoclypeal area triangular, lateral disc with sparse punctures and setae; labrum transverse, with sparse setae; antennae nearly 1/2 of body length, antennomeres 1–4 nearly globular, antennomere 2 shortest, antennomeres 5–10 nearly 1.5× as long as wide, antennomere 11 slender.

***Pronotum*** (Figs 7, 38B). Anterior angles slightly protruding, posterior angles not protruding; sides constricted in the middle; disc slightly raised; middle of disc with two or three irregular rows fine punctures.

***Elytra*** (Figs 7, 28B). Humeri protruding, humeral groove and basal impression distinct; strial punctures large in the base, diminishing posteriorly, absent on apical 1/4; intervals smooth; epipleura raised, with a sparse row of fine punctures.

***Mesosternum pubescent*.** Lateral metasternum with an arcuate strip of pubescence, extending from anterior margin to lateroposterior corner. Metepisternum densely pubescent (Fig. 38C).

***Abdominal sternite*** (Fig. 28A). Lateral transverse impressions big and distinct on sternites 1–3, other areas of sternites 1–3 and all of sternites 4 and 5 densely pubescent.

***Leg*** (Fig. 28C). Femora with dense pubescence on the dorsal surface, nearly smooth on the ventral surface, femora of hind legs with a distinct tooth, and middle legs with a weak tooth.

***Male genitalia*** (Fig. 47A–D). Ostium occupying 1/5 length of median lobe (Fig. 47A); apex round (Fig. 47B); tegmen Y-shaped, basal piece of tegmen triangular and narrow, lateral lobes slightly sclerotized and combined with second connecting membrane; internal sac membranous, with distinct dorsal, median, and ventral sclerites, posterior part of dorsal sclerite in dorsal view slightly widen, ventral sclerite short and flat, median sclerite distinct (Fig. 47C, D).

***Female reproductive organs*** (Fig. 57A–C). Tergites 8 and 9, sternites 8 and 9 sclerotized, posterior areas of tergite 8, sternite 8, and apodemes with pubescence, spiculum gastrale Y-shaped and long; ovipositor with dense pubescence, distal part of ovipositor cylindrical, short, and with a protuberance; spermatheca simple and hooked.

##### Distribution

**(Fig. 68).** China (Hainan); Vietnam.

##### Host plant and habitat.

Host plant is unknown. We visited Jianfengling of Hainan where all Chinese specimens were collected, located in a subtropical area. The habitat is mixed primeval forest, orchards, and farmland with high temperatures, plentiful precipitation, and plenty of sunlight. The type locality, Tianchi, refers to a lake area surrounded by shrubs and tall trees.

##### Remarks.

*Liliocerisdentifemoralis* can be distinguished from other species in this group by the femora of mid- and hind legs with a tooth (Fig. 32C). This species seems to be very rare: we tried several times to collect this species in Hainan but failed.

#### ﻿ Lilioceris discrepens

Taxon classificationAnimaliaColeopteraChrysomelidae

(Baly, 1879)

F6B10B90-F5F7-5A88-9AF3-DECDEEAE4386

Figs 9 , 10 , 29A–D , 39A–C , 51A–D , 65A–C , 67 , 70A, B

Crioceris discrepens Baly, 1879: 316 (Siam, Laos).
Lilioceris discrepens : Kimoto and Gressitt 1979: 220.

##### Type material examined.

***Syntype*** of *Liliocerisdiscrepens* (NHML, photo), Type / Type / Baly Coll. / Criocerisdiscrepens Baly, Siam / Criocerisdiscrepens Baly, Type / BMNH(E)1344920.

##### Other material examined.

Total 29 specimens. **China: Yunnan**: 1♂, Gongshan, Dulongjiang, Maku, 2021.5.31 / 27.68936°N, 98.30804°E, 1691 m, Liang H.B., Xu Y., Zhang N. coll.; 1♀, Jinping, Jinzhuzhai, 2012. 5.14, Yang XD coll.; 1♀1♂, Xinping, Ailao Shan, 2021.5.16 / N24.306612, E101. 351084, 1732 m, Zhang N. coll; 1♂, Ruili, Bangdazhai, N. Y. Tsa coll.; 1♀, Gongshan, Dulongjiang, Qinlandang, beach. 27.67854°N, 98.28731°E, 1970 m, 2006.8.31 day, Liu Ye collector California Academy and IOZ. Chinese. Acad. Sci., L.discrepens, det. Liang H.B. 2019; 1♀, Xishuangbanna, Mengla, Longlin Xincun, 21.52914°N, 101.49415°E, 1066 m / Hongbin Liang and Yuan Xu coll.; 1♀, Malipo, Lao Shan Yaowanggu, 2021.4.19 / 23.00919°N, 104.82562°E, 1050 m, Hongbin Liang, Yuan Xu and Neng Zhang coll.; 1♀, Xishuangbanna, Menghai, Bulang Shan, 2011.4.28, Hongbin Liang and Kaiqin Li coll.; 2♀, Xishuangbanna, Mengla, 620–650 m, 1958.XI.16, Yiran Zhang coll.; 1♀, Jinping, Changpotou, 1000 m, 1956.V.22, Keren Huang coll.; 2♀, Xishuangbanna, Mengla, 620–650 m, 1959.VII.7, Facai Zhang coll.; 1♀, Xishuangbanna, Menga, 1050–1080 m, 1958.V.17, Fuji Pu coll.; 1♀, Jinping, Changpotou, 1200 m, 1956.V.23, Keren Huang coll.; 1♀, Xishuangbanna, Menglun botanic garden, 1988.XI.20; 1♂, Xishuangbanna, Mengla, 620–650 m, 1959.VI.2, Yiran Zhang coll.; 1♂, Xishuangbanna, Mengla, 620–650 m, 1959.V.29, Suofu Li coll.; 1♂, Ruili, Bangdazhai, 2014.IX.4, NY Tsa coll.; 1♀, Xishuangbanna, Mengla, Longlin New village, 21.52914°N, 101.49415°E, 1066 m, 2020.VI. 6, Hongbin Liang and Yuan Xu coll.; 1♂, Gongshan, Dulongjiang, Maku village, 27.68979°N, 98.30513°E, 1733 m, 2021.V.31, Hongbin Liang and Yuan Xu coll.; 1♀, Jinping, Jinzhuzhai, 2012.V.14, Xiaodong Yang coll.; 1♀, Malipo, Lao Shan Yaowanggu, 23.00919°N, 104.82562°E, 1050 m, 2021.IV.19, Hongbin Liang and Yuan Xu coll.; 1♀1♂, Xinping, Dacunzi, 24.306612°N, 101.351084°E 1731 m, 2021.V.16, Neng Zhang coll.; **Guangxi**: 1♂, Guangxi, Longzhou, Daqing Shan, 360 m, 1963.IV.23, Chunguang Wang coll.; **Vietnam**: 1♂, Museum Paris, Tonkin N., Reg Dha-Giang (H Riviere Claire) Siebens Olivier 1916.

##### Diagnosis.

Anterior angles of pronotum protruding, pronotal disc with four or five irregular rows of fine punctures. Humeral groove of elytra distinct, punctures of elytra sparse and diminishing posteriorly, absent on apical 1/3. Femora of mid- and hind legs without tooth. Lateral metasternum with a long strip of pubescence.

##### Redescription.

BL = 9.5–14.5 mm, BW = 4.4–4.7 mm. ***Body*** brownish red.

***Head*** (Figs 9, 39A). Vertex flat, with a deep groove in the middle, sparsely punctate and pubescent laterally; frontoclypeal area triangular, lateral disc with sparse punctures and pubescence; labrum transverse, with sparse pubescence; antennae nearly 1/2 of body length, antennomeres 1–4 nearly globular, antennomere 2 shortest, antennomeres 5–10 nearly 1.1× as long as wide, antennomere 11 slender.

***Pronotum*** (Figs 9, 39B). Anterior angles protruding, posterior angles not protruding; sides slightly constricted in the middle; disc flat; middle of disc with four or five irregular rows of fine punctures. Scutellum triangular and pubescent.

***Elytra*** (Figs 9, 29B). Humeri protruding, humeral groove and basal impression distinct; elytra without completely punctate striae, punctures sparse and large in the base, diminishing posteriorly, absent on apical 1/3; intervals smooth; epipleura raised, with a sparse row of fine punctures.

***Mesosternum pubescent*.** Lateral metasternum with a long and arcuate strip of pubescence, metepisternum densely pubescent. Metepisternum densely pubescent (Fig. 39C).

***Abdominal sternite*** (Fig. 29A). Lateral transverse impressions big and distinct on sternites 1–4, other areas of sternites 1–4 densely pubescent.

***Leg*** (Fig. 29C). Femora with dense pubescence on the dorsal surface, with sparse pubescence on the ventral surface, without tooth.

***Male genitalia*** (Fig. 51A–D). Ostium occupying 1/4 length of median lobe (Fig. 51A); apex sharp (Fig. 51B); tegmen Y-shaped, basal piece of tegmen triangular and broad, lateral lobes sclerotized and combined with second connecting membrane; internal sac membranous, with distinct dorsal and ventral sclerites, posterior part of dorsal sclerite in dorsal view parallel, ventral sclerite elongated and curly (= flagellum), median sclerite small (Fig. 51C, D).

***Female reproductive organs*** (Fig. 65A–C). Tergites 8 and 9, sternites 8 and 9 sclerotized, posterior areas of tergite 8, sternite 8, and apodemes with pubescence, spiculum gastrale Y-shaped and long; ovipositor with dense pubescence, distal part of ovipositor cylindrical, long and with a protuberance; spermatheca simple and hooked.

##### Distribution

**(Fig. 67).** China (Yunnan, Guangxi); Laos; Thailand; Vietnam. New record from China

##### Host plant and habitat

**(Fig. 70A, B).** This species is fed on *Smilax* sp. according to our observations in Yunnan. One locality of this species in Maku village (Yunnan, Gongshan, Dulongjiang) is situated at the subtropics. The habitat is primeval forest, which is characterized by both high temperatures and humidity, plentiful precipitation, but without much sunlight. The forests are composed of tall trees, woody vines, and weeds.

##### Remarks.

The body size of this species is very large for the genus. It is similar to *L.latissima*, but differs by the pronotal disc with four or five irregular rows of fine punctures, and the lateral metasternum with a long arcuate strip of pubescence (in *L.latissima*, the pronotal disc has two rows of fine punctures, and the lateral metasternum is smooth).

*Liliocerisdiscrepens* was described by Baly (1879) from Laos, subsequently listed with simple characteristics in a key by Kimoto and Gressitt (1979: 220) and an illustration of the sternum and episternum (Kimoto and Gressitt 1979: 224, fig. 14j), but we examined the types (Figs 9, 10) and found that the identification was wrong. The lateral metasternum has a long strip of pubescence, rather than the sparse pubescence as illustrated by Kimoto and Gressitt (1979).

#### ﻿ Lilioceris jianfenglingensis

Taxon classificationAnimaliaColeopteraChrysomelidae

Long, 1988

D8A67B27-33BA-501D-91CF-3C732808CFDD

**Figures 11–14.**
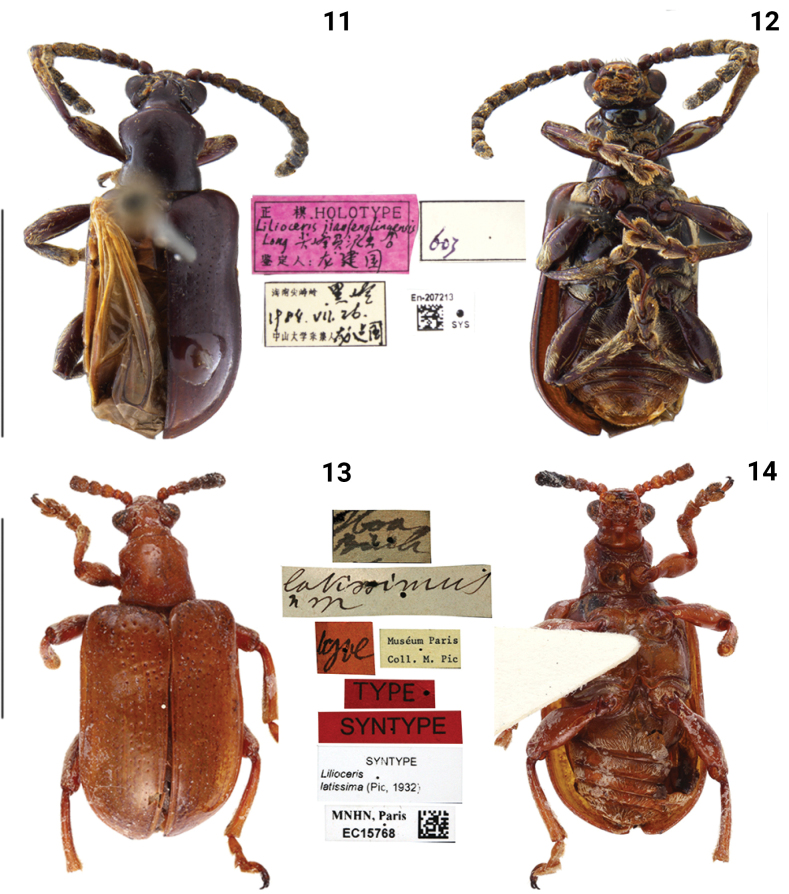
Habitus of *Lilioceris* spp. **11, 12***L.jianfenglingensis*, holotype, China (Hainan), photographed by Yuan Xu **13, 14***L.latissima*, syntype, Vietnam (Tonkin), photographed by Christophe Rivier (MNHN). Scale bars: 5.0 mm.

Figs 11 , 12 , 30A–D , 40A–C , 54A–D , 61A–C , 68

Lilioceris jianfenglingensis Long, 1988: 231 (China: Hainan).

##### Type material examined.

***Holotype*** of *Liliocerisjianfenglingensis* (MBSU), Hainan, Jianfengling, Heiling, 1984.VII.26, Jianguo Long coll. / holotype, Liliocerisjianfenglingensis Long ♂, Jianguo Long det. / 603 / En–207213 SYS; ***allotype*** of *Liliocerisjianfenglingensis* (MBSU), Hainan, Jianfengling, Zhufeng, 1412.5 m, 1982.2.27, Yongcheng Long coll. / Allotype, Liliocerisjianfenglingensis Long ♀, Jianguo Long det. / En–207212 SYS; ***paratype*** of *Liliocerisjianfenglingensis* (MBSU), Hainan, Jianfengling, Fifth Area, (14)55, 1991.VII.7, Jiadong Chen coll. / En–207211 SYS / Liliocerissemipunctata ? (F. 1801), det. Liang H.B. 2004; 1♂, Hainan, Jianfengling, Fifth Area, 1982.2.22, Yongcheng Long coll. / Liliocerisjianfenglingensis Long, Jianguo Long det. ♂ / En–207210 SYS.

##### Other material examined.

Total 38 specimens. **China: Yunnan**: 2♂, Xishuangbanna, Menga,1050–1080 m, 1958.V.13, Shuyong Wang coll.; 1♂, Xishuangbanna, Damenglong, 1050–1080 m, 1958.IV.24, Yiran Zhang coll.; 1♂, Xishuangbanna, Menghun, 1200–1400 m, 1958.IV.12, Chunpei Hong coll.; 1♂, Xishuangbanna, Mengsong, 1600 m, 1958.IV.24, Fuji Pu coll.; 1♂, Xishuangbanna, Mengsong, 1600 m, 1958.IV.24, Fuji Pu coll.; 1♂, Xishuangbanna, Mengzhe, 1200 m, 1958.VI.14, Shuyong Wang coll.; 1♂, Xishuangbanna, Mengsong, 1600 m, 1959.IV.24, Chunpei Hong coll.; 1♂, Xishuangbanna, Menghun, 1200–1400 m, 1959.IV.24, Chunpei Hong coll.; 1♂, Xishuangbanna, Menghun, 1200–1400 m, 1958.V.10, Leyi Zheng coll.; 1♂, Xishuangbanna, Mengsong, 1600 m, 1959.VII.26.; 1♂, Xishuangbanna, Mengsong, 1600 m, 1958.IV.22, Shuyong Wang coll.; 1♂, Xishuangbanna, Menghai, 1200–1600 m, 1957.VIII.10, Lingchao Zang coll.; 1♂, Xishuangbanna, Menga,1050–1080 m, 1958.V.17, Fuji Pu coll.; 1♂, Xishuangbanna, Xiaomengyang, 850 m, 1958.VIII.31; 1♂, Xishuangbanna, Mengsong, 1600 m, 1958.IV.27, Xuwu Meng coll.; 1♂, Xishuangbanna, Menga,1050–1080 m, 1958.VI.1, Fuji Pu coll.; 1♂, Xishuangbanna, Damenglong, 650 m, 1958.IV.8, Fuji Pu coll.; 1♂, Xishuangbanna, Menghun, 1200–1400 m, 1958.V.10, Chunpei Hong coll.; 1♂, Xishuangbanna, Menga,1050–1080 m, 1958.VIII.4, Fuji Pu coll.; 1♀, Xishuangbanna, Menghun, 1200 m, 1958.V.28, Yiran Zhang coll.; 1♀, Xishuangbanna, Menga,100 m, 1958.V.23, Fuji Pu coll.; 1♀, Xishuangbanna, Menga, 1050–1080 m, 1958.X.19, Zhizi Chen coll.; 1♀, Xishuangbanna, Mengla, 620–700 m, 1959.V.29, Yiran Zhang coll.; 1♀, Xishuangbanna, Menga,1050 m, 1958.V.17, Fuji Pu coll.; 1♀, Xishuangbanna, Menga, 1050–1080 m, 1958.VIII.4, Shuyong Wang coll.; 1♀, Xishuangbanna, Mengyang, 620 m, 1981.IV.12, Fasheng Li coll.; 1♀, Xishuangbanna, Menga, 1050 m, 1958.V.23, Fuji Pu coll.; 1♀, Xishuangbanna, Menghai, 1200 m, 1957.IV.24, Yiran Zhang coll.; 1♀, Xishuangbanna, Menga, 1050–1080 m, 1958.V.17, Shuyong Wang coll.; 1♀, Xishuangbanna, Mengsong, 1600 m, 1958.IV.24, Fuji Pu coll.; 1♀, Xishuangbanna, Menga, 1050–1080 m, 1958.VIII.4, Shuyong Wang coll.; 1♀, Xishuangbanna, Menghun, 1200 m, 1958.V.10, Yiran Zhang coll.; 1♀, Xishuangbanna, Mengsong, 1600 m, 1958.VIII.18, Fuji Pu coll.; 1♀, Xishuangbanna, Menga, 1050–1080 m, 1958.VIII.22, Shuyong Wang coll.; 1♀, Xishuangbanna, Mengzhe, 1700 m, 1958.VI.23, Zhizi Chen coll.; 1♀, Xishuangbanna, Menga, 1050–1080 m, 1958.V.25, Shuyong Wang coll.; 1♀, Xishuangbanna, Menga, 1050–1080 m, 1958.VIII.18, Fuji Pu coll.

##### Diagnosis.

Antennomeres 5–10 as long as wide, flattened. Anterior angles of pronotum protruding, pronotal disc with two rows of fine punctures. Humeral groove of elytra indistinct, punctures sparse and diminishing posteriorly, absent on apical 1/3. Lateral metasternum with a short strip of pubescence.

##### Redescription.

BL = 7.5–9.5 mm, BW = 3.5–4.0 mm. ***Body*** brownish red.

***Head*** (Figs 11, 40A). Vertex flat, with a very shallow groove in the middle, sparsely punctate and pubescent laterally; frontoclypeal area triangular, lateral disc with sparse punctures and pubescent; labrum transverse, with sparse pubescent; antennae nearly 1/2 of body length, antennomeres 1–4 nearly globular, antennomere 2 shortest, antennomeres 5–10 as long as wide, antennomere 11 slender.

***Pronotum*** (Figs 11, 40B). Anterior angles protruding, posterior angles not protruding; sides distinctly constricted in the middle; disc flat; middle of disc with two rows fine punctures. Scutellum triangular and pubescent.

***Elytra*** (Figs 11, 30B). Humeri protruding, humeral groove and basal impression shallow; elytra without completely punctate striae, punctures sparse, diminishing posteriorly, absent on apical 1/3; intervals smooth; epipleura raised, with a row of fine punctures.

***Mesosternum pubescent*.** Lateral metasternum with oblique strip of pubescence. Metepisternum densely pubescent (Fig. 40C).

***Abdominal sternite*** (Fig. 30A). Lateral transverse impressions big and distinct on sternites 1–4, other areas of sternites 1–4 densely pubescent.

***Leg*** (Fig. 30D). Femora with dense pubescence on the dorsal surface, with sparse pubescence on the ventral surface, without tooth.

***Male genitalia*** (Fig. 54A–D). Ostium occupying 1/2 length of median lobe (Fig. 54A); apex sharp (Fig. 54B); tegmen Y-shaped, basal piece of tegmen oval and narrow, lateral lobes slightly sclerotized and combined with second connecting membrane; internal sac membranous, with distinct ventral sclerites, dorsal sclerite nearly membranous, ventral sclerite greatly long and tubular (= flagellum), median sclerite small (Fig. 54C, D).

***Female reproductive organs*** (Fig. 61A–C). Tergites 8 and 9, sternites 8 and 9 sclerotized, posterior areas of tergite 8, sternite 8, and apodemes with pubescence, spiculum gastrale Y-shaped and long; ovipositor with dense pubescence, distal part of ovipositor cylindrical, long and with a protuberance; spermatheca simple and hooked.

##### Distribution

**(Fig. 68).** China (Hainan, Yunnan).

##### Host plant and habitat.

Unknown.

##### Remarks.

The species is most similar to *L.yuae*, but differs by the antenna being brownish red, without a metallic luster, and the antennomeres 7–10 as long as wide (Fig. 30D); the lateral transverse impressions are larger on the abdominal sternites 1–4 (Fig. 30A); the ventral sclerite is long and thin (Fig. 54C, D). (in *L.yuae*, the antenna is brownish red with a blue metallic luster, antennomeres 7–10 are distinctly longer than wide (Fig. 36D); lateral transverse impressions are very small on all sternites (Fig. 36A); and the ventral sclerite is shorter and thicker (Fig. 55C, D)).

#### ﻿ Lilioceris latissima

Taxon classificationAnimaliaColeopteraChrysomelidae

(Pic, 1932)

A5684AA0-C638-5F94-9D82-5FEE3BEE6927

Figs 13 , 14 , 31A–D , 41A–C , 53A–D , 60A–C , 67

Crioceris latissimus Pic, 1932: 10 (Vietnam: Tonkin).
Lilioceris latissima : Kimoto and Gressitt 1979: 227.

##### Type material examined.

***Syntype*** of *Liliocerislatissimus* (MNHN, photo), Crioceris latissimi Pic / Type / Museum Paris, Coll. M. Pic / Type / Syntype / Syntype, Lilioceris latissimi (Pic, 1932) / MNHN, Paris EC15768.

##### Other material examined.

Total 96 specimens. **China: Yunnan**: 1♀, Xishuangbanna, Mengla, 620–650 m, 1959.V.29, Suofu Li coll.; 1♀1♂, Xishuangbanna, Menglun, 650 m, 1959.VIII.3, Fuji Pu coll.; 1♀, Xishuangbanna, Damenglong, 650 m, 1958.VIII.8, Yiran Zhang coll.; 1♀, Xishuangbanna, Mengla, 620–650 m, 1959.VI.8, Facai Zhang coll.; 1♀, Lancang, 1200 m, 1957.VII.30, Lingchao Zang coll.; 1♀, Xishuangbanna, Mengla, 700 m, 1959.V.19, Fuji Pu coll.; 1♀, Xishuangbanna, Mengzhe, 870 m, 1958.VII.9, Shuyong Wang coll.; 1♀, Xishuangbanna, Jinghong, 650 m, 1959.VI.8, Xuezhong Zhang coll.; 1♀, Xishuangbanna, Mengzhe, 870 m, 1958.VII.11, Fuji Pu coll.; 1♀, Xishuangbanna, Mengla, 620–650 m, 1959.VI.6, Yiran Zhang coll.; 1♀, Xishuangbanna, Mengla, 620–650 m, 1959.VII.8, Suofu Li coll.; 1♀, Xishuangbanna, Xiaomengyang, 850 m, 1958.IX.1, Xuwu Meng coll.; 1♀, Xishuangbanna, Mengzhe, 620–650 m, 1959.V.9, Facai Zhang coll.; 1♀, Xishuangbanna, Mengla, 620–650 m, 1959.VI.30, Facai Zhang coll.; 2♀, Xishuangbanna, Menghun, 1200–1400 m, 1958.VI.13, Yiran Zhang coll.; 1♀, Xishuangbanna, Xiaomenghun, 650–750 m, 1958.VI.13, Xuwu Meng coll.; 1♀, Xishuangbanna, Menglun, 650 m, 1959.VIII.27, Fuji Pu coll.; 1♀, Xishuangbanna, Mengla, 620–650 m, 1959.VII.13, Suofu Li coll.; 1♀, Xishuangbanna, Menghai, Nannuo Shan, 1100–1500 m, 1957.IV.27, Fuji Pu coll.; 1♀, Jinping, Mengla, 500 m, 1956.IV.20, Keren Huang coll.; 1♀, Xishuangbanna, Mengla, 620–650 m, 1959.VII.8, Facai Zhang coll.; 2♀, Xishuangbanna, Menglun, 650 m, 1959.VII.24, Facai Zhang coll.; 1♀, Xishuangbanna, Menghun, 750 m, 1958.VI.5, Xuwu Meng coll.; 1♀, Xishuangbanna, Menghai, 1400 m, 1980.III.10, Zhiming Li coll.; 1♀, Xishuangbanna, Menglun, 650 m, 1958.IX.29, Shuyong Wang coll.; 1♀, Xishuangbanna, Mengla, 620–650 m, 1959.VII.8, Facai Zhang coll.; 1♀, Xishuangbanna, Mengla, 620–650 m, 1959.V.29, Suofu Li coll.; 1♀, Jinghong, 1984.IV.20; 1♀, Ruili, Ruili Botanical garden, N 24.07230, E 97.81944 / 2012.X.27, 1152 m, Huang Xinle Leg. Inst. of Zoology, CAS; 1♂, Cheli [= Jinghong], 580 m, 1957.III.10, Fuji Pu coll.; 2♂, Xishuangbanna, Mengla, 620–650 m, 1959.VI.8, Suofu Li coll.; 1♂, Xishuangbanna, Menglun, 650 m, 1959.VII.24, Yiran Zhang coll.; 1♂, Xishuangbanna, Xiaomengyang, 850 m, 1958.IX.13, Yiran Zhang coll.; 1♂, Xishuangbanna, Menglun, 650 m, 1959.VII.31, Yiran Zhang coll.; 2♂, Xishuangbanna, Mengla, 620–650 m, 1959.VI.2, Fuji Pu coll.; 1♂, Xishuangbanna, Mengla, 620–650 m, 1959.VII.8, Fuji Pu coll.; 1♂, Xishuangbanna, Mengla, 620–650 m, 1959.VII.13, Suofu Li coll.; 2♂, Xishuangbanna, Xiaomengyang, 850 m, 1958.IX.1, Xuwu Meng coll.; 1♂, Xishuangbanna, Mengla, 620–650 m, 1959.V.19, Fuji Pu coll.; 1♂, Xishuangbanna, Mengla, 620–650 m, 1959.V.19, Facai Zhang coll.; 1♂, Xishuangbanna, Menghun,1200–1400 m, 1958.VI.13, Yiran Zhang coll.; 1♂, Xishuangbanna, Mengla, 620–650 m, 1959.VI.5, Fuji Pu coll.; 1♂, Xishuangbanna, Menglun, 650 m, 1959.VII.24, Yiran Zhang coll.; 1♂, Xishuangbanna, Xiaomengyang, 850 m, 1957.IX.6, Lingchao Zang coll.; 1♂, Xishuangbanna, Mengla, 700 m, 1959.V.19, Fuji Pu coll.; 1♂, Xishuangbanna, Mengla, 700 m, 1959.V.8, Fuji Pu coll.; 1♂, Xishuangbanna, Menglun, 650 m, 1959.VII.27, Fuji Pu coll.; 1♂, Xishuangbanna, Menglun, 650 m, 1959.VII.30, Fuji Pu coll.; 1♂, Xishuangbanna, Menglun, 620–650 m, 1959.VII.8, Fuji Pu coll.; 1♂, Xishuangbanna, Mengzhe, 1200 m, 1958.IX.14, Shuyong Wang coll.; 1♂, Xishuangbanna, Mengla, 620–650 m, 1959.V.14, Facai Zhang coll.; 1♂, Xishuangbanna, Damenglong, 650 m, 1958.VIII.8, Yiran Zhang coll.; 1♂, Xishuangbanna, Menglun, 650 m, 1959.VIII.3, Suofu Li coll.; 1♂, Xishuangbanna, Menglun, 650 m, 1959.VIII.25, Suofu Li coll.; 1♂, Xishuangbanna, Menglun, 650 m, 1959.VIII.3, Facai Zhang coll.; 1♂, Xishuangbanna, Menglun, 650 m, 1958.IX.29, Shuyong Wang coll.; 1♂, Damenglong, 700 m, 1957.IV.12, Shuyong Wang coll.; 1♂, Jinping, Mengla, 500 m, Keren Huang coll.; 1♂, Xishuangbanna, Menglun, 650 m, 1959.VIII.3, Fuji Pu coll.; 1♂, Xishuangbanna, Menglun, 650 m, 1959.VII.27, Fuji Pu coll.; 1♂, Xishuangbanna, Xiaomengyang, 850 m, 1958.X.20, Fuji Pu coll.; 1♂, Xishuangbanna, Damenglong, 650 m, 1958.IV.9, Fuji Pu coll.; 1♂, Xishuangbanna, Xiaomengyang, 850 m, 1958.IX.6, Yiran Zhang coll.; 1♂, Xishuangbanna, Mengzhe, 1200 m, 1958.IX.14, Shuyong Wang coll. 1♂, Xishuangbanna, Xiaomengyang, 850 m, 1958.VII.24, Yiran Zhang coll.; 1♂, Jinghong, 1984.IV.19; 1♀, Xishuangbanna, Mengyang, Baihua Shan, 22.17720°N, 100.92428°E, 876 m, 2020.VI.3, Hongbin Liang and Yuan Xu coll.; 1♀1♂, Xishuangbanna, Mengyang, Baihua Shan, 22.17720°N, 100.92428°E, 876 m, 2021.IV.3, Hongbin Liang, Yuan Xu and Neng Zhang coll.; 2♀3♂, Jinping, Mengla, Wengdang village, 2011.IV.17, Hongbin Liang and Kaiqin Li coll.; 1♀, Tengchong, Datang, 2014.VIII.3, Hongbin Liang coll.; **Guangxi**: 1♂, Baishou, 1952.6.25; **Vietnam**: 1♂, Tonkin, Hoa Binh, 1939.V.II, leg. A de Cooman; 2♂, Tonkin, Hoa Binh, 1940.V.III, leg. A de Cooman; 2♂, Tonkin, Hoa Binh, leg. A de Cooman; 2♂, Tonkin; 1♂, Museum Paris, Cochinchine, Amiral Vicnes, 1898; 1♀, Tonkin, Hoa Binh, 1939.V.II, leg. A de Cooman; 1♀, Tonkin, Hoa Binh, 1940.V.III, leg. A de Cooman; 1♀, Tonkin; 2♀, Tonkin, Hoa Binh, leg. A de Cooman.

##### Diagnosis.

Anterior angles of pronotum protruding, pronotal disc with two rows of fine punctures. Humeral groove of elytra indistinct, punctures sparse and diminishing posteriorly, absent on apical 1/3. Metasternum smooth.

##### Redescription.

BL = 7.7–8.9 mm, BW = 3.0–4.0 mm. ***Body*** brownish red.

***Head*** (Figs 13, 41A). Vertex flat, with a shallow groove in the middle, sparsely punctate and pubescent laterally; frontoclypeal area triangular, lateral disc with sparse punctures and pubescent; labrum transverse, with sparse pubescent; antennomeres 5–10 almost as long as wide, flattened.

***Pronotum*** (Figs 13, 41B). Anterior angles protruding, posterior angles slightly protruding; sides slightly constricted in the middle; disc flat; middle of disc with three or four rows of fine and shallow punctures. Scutellum triangular and pubescent.

***Elytra*** (Figs 13, 31B). Humeri protruding, humeral groove and basal impression shallow; elytra without completely punctate striae, punctures sparse, diminishing posteriorly, absent on apical 1/3; intervals without punctures; epipleura raised, with a row of fine punctures.

***Mesosternum pubescent*.** Metasternum smooth. Metepisternum densely pubescent (Fig. 41C).

***Abdominal sternite*** (Fig. 31A). Lateral transverse impressions distinct on sternites 1–4, other areas of sternites 1–4 densely pubescent.

***Leg*** (Fig. 31C). Femora with dense pubescence on the dorsal surface, with sparse pubescence on the ventral surface, without tooth.

***Male genitalia*** (Figs 53A–D). Ostium occupying 1/3 length of median lobe (Fig. 53A); apex hooked (Fig. 53B); tegmen Y-shaped, basal piece of tegmen triangular and broad, lateral lobes slightly sclerotized and combined with second connecting membrane; internal sac membranous, with distinct dorsal and ventral sclerites, posterior part of dorsal sclerite in dorsal view round, long and tubular (= flagellum), median sclerite small.

***Female reproductive organs*** (Fig. 60A–C). Tergites 8 and 9, sternites 8 and 9 sclerotized, posterior areas of tergite 8, sternite 8, and apodemes with pubescence, spiculum gastrale Y-shaped and long; ovipositor with dense pubescence, distal part of ovipositor cylindrical, long and with a protuberance; spermatheca convoluted.

##### Distribution

**(Fig. 67).** China (Yunnan; Guangxi); Vietnam. New record from China.

##### Host plant and habitat.

This species is fed on *Smilaxcorbularia* Kunth according to our observation in Yunnan. One locality of this species in Baihua Shan (Yunnan, Xishuangbanna, Jinghong) is situated at subtropics. This habitat is mixed primeval forest and farmland, with high temperatures, plentiful precipitation, and plenty of sunlight.

##### Remarks.

*Liliocerislatissima* can be distinguished from other species in this group by the smooth metasternum (Fig. 41C) and middle of pronotal disc with three or four rows of fine punctures (Fig. 41B).

This species was described by Pic (1932) from Tonkin, Vietnam. Subsequently, Kimoto and Gressitt listed simple characteristics in a key (1979: 220) and provided an illustration for the sternum and episternum (1979: 224, fig. 141). We examined the types (Figs 13, 14) and found that their identification was wrong: the lateroposterior corner of the metasternum of the type is glabrous, rather than densely pubescent as illustrated by Kimoto and Gressitt (1979). Furthermore, the distributions in Thailand and Laos listed by Kimoto and Gressitt need confirmation, and we therefore exclude them in this study.

#### ﻿ Lilioceris lianzhouensis

Taxon classificationAnimaliaColeopteraChrysomelidae

Long, 2000a

63BD1CF0-7F58-5AB0-AE51-FB2ECEF99283

**Figures 15–18.**
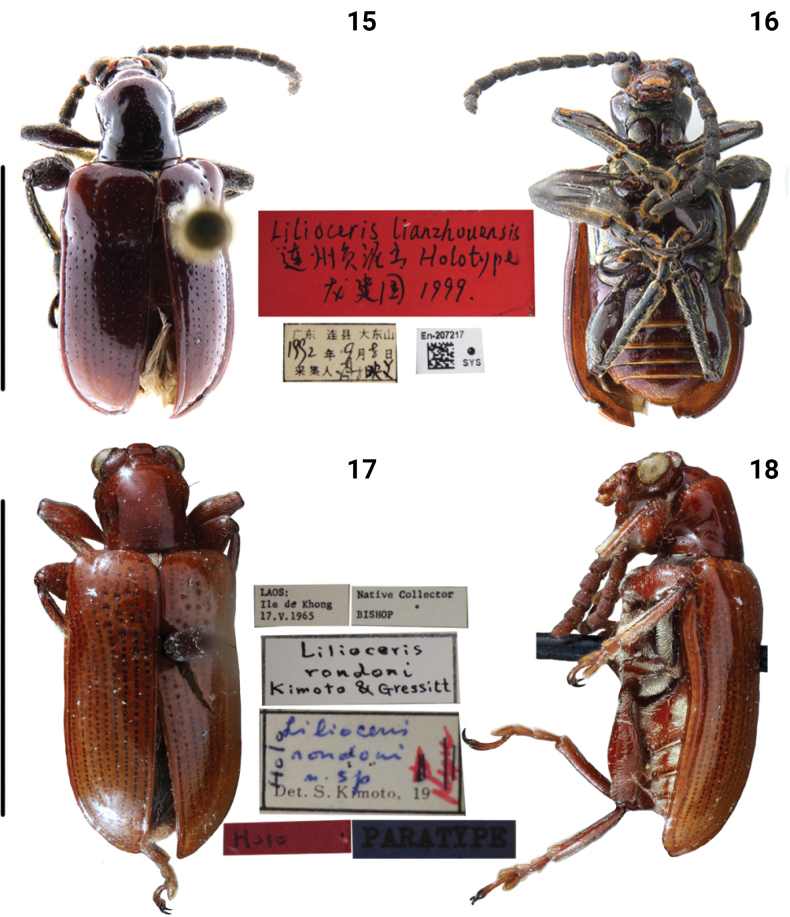
Habitus of *Lilioceris* spp. **15, 16***L.lianzhouensis*, holotype, China (Guangdong), photographed by Yuan Xu **17, 18***L.rondoni*, holotype, Laos (Sithandone), photographed by Jeremy Frank (BSM). Scale bars: 5.0 mm.

Figs 15 , 16 , 32A–D , 42A–C , 52A–D , 59A–C , 68

Lilioceris lianzhouensis Long, 2000a: 262 (China: Guangzhou).

##### Type material examined.

***Holotype*** of *Liliocerislianzhouensis* (MBSU), Liliocerislianzhouensis, Holotype, Jianguo Long det., 1999 / Guangdong, Lianzhou County, Dadong Shan, 1992.9.8, Yingwen Xie coll. / EN–207217 SYS; ***allotype*** of *Liliocerislianzhouensis* (MBSU), Liliocerislianzhouensis, Allotype, Jianguo Long det. / Guangdong, Lianxian, Dadong Shan, 1996.8.26, Weicai Xie coll. / EN–207216 SYS.

##### Other material examined.

Total 18 specimens. **China: Hunan**: 1♂, Yizhang, Mangshan Park, near Nanling, 24.95127°N, 112.98377°E / 1339 m, 2008.7.19, Ganyan Yang coll., Institute of Zoology; 1♀, Mangshan, 2021.VI.27, Yong Wang coll.; **Yunnan**: 1♂, Yuanyang, Shangxincheng, 2022.IV.22, Neng Zhang coll.; 1♀, Lvchun, Huanglian Shan, 2018.V.23, Kaiqin Li coll.; **Guangxi**: 2♀1♂, Nanning, Daming Shan Nature Reserve, 2011.5.20–23, Kaiqin Li coll.; 1♂, Nanning, Daming Shan Daxiagu, on vegetation, 23.49960°N, 108.42891°E / 1111 m, 2011.5.27, Kaiqin Li coll., Institute of Zoology, CAS; 1♂, Nanning, Daming Shan Nature Reserve, Tianping / 1230 m, 2011.V.29, Kaiqin Li coll., Inst. of Zoology, CAS; 1♀, Longsheng, Huaping, Anjiangping, 2006.VIII.3, Meiying Lin coll.; **Guangdong**: 1♀, Dinghu Shan, 1979.IV.16–20, Shaokun Du and Jinying Liang coll.; 1♀, Shikengkong, 1300 m, 1995.VII.22, W. Lu coll.; 1♀, Shenzhen, Dapeng peninsula, N22.82931, 114.52472 / 5m, 2018.VII.19, Hongbin Liang and Yuan Xu coll.; **Hainan**: 1♂, Baisha, Hongkan Reservior, 19.08121°N, 109.49839°E / 525 m, 2009.11.24, Hongbin Liang coll., Institute of Zoology; 1♂, Qiongzhong, Wuzhi Shan, 800 m, 1980.IV.5, Fuji Pu coll.; 2♀1♂, Baisha, Gaofeng village, 19.04059°N, 109.31583°E, 886 m, 2020.7.28, Yuan Xu coll.

##### Diagnosis.

Anterior angles of pronotum protruding, pronotal disc with two or three irregular rows of fine punctures. Humeral groove of elytra shallow, punctures of elytra diminishing posteriorly, absent on apical 1/3. Femora of legs without tooth. Lateral metasternum with a long and arcuate strip of pubescence.

##### Redescription.

BL = 8.7–11.2 mm, BW = 3.0–4.5 mm. ***Body*** brownish red, head, antenna, legs and lateral metasternum with a blue metallic luster.

***Head*** (Figs 15, 42A). Vertex flat, with a shallow groove in the middle, sparse punctate and pubescent laterally; frontoclypeal area triangular, lateral disc with sparse punctures and pubescent; labrum transverse, with sparse pubescent; antennae nearly 1/2 of body length, antennomeres 1–4 nearly globular, antennomere 2 shortest, antennomeres 5–10 nearly 1.5× as long as wide, antennomere 11 slender.

***Pronotum*** (Figs 15, 42B). Anterior angles slightly protruding, posterior angles not protruding; sides constricted in the middle; disc slightly raised; middle of disc with two or four irregular rows of fine punctures.

***Elytra*** (Figs 15, 32B). Humeri protruding, humeral groove and basal impression distinct; elytra without completely punctate striae, punctures large in the base, diminishing posteriorly, absent on apical 1/4; intervals smooth; epipleura raised, with a sparse row of fine punctures.

***Mesosternum pubescent*.** Lateral metasternum with a long and arcuate strip of pubescence, extending from anterior margin to lateroposterior corner, metepisternum densely pubescent (Fig. 42A).

***Abdominal sternite*** (Fig. 32A). Lateral transverse impressions big and distinct on sternites 1–4, other areas of sternite densely pubescent.

***Leg*** (Fig. 32C). Femora with dense pubescence on the dorsal surface, nearly smooth on the ventral surface, without tooth.

***Male genitalia*** (Fig. 52A–D). Ostium occupying 1/3 length of median lobe (Fig. 52A); apex crooked (Fig. 52B); tegmen Y-shaped, basal piece of tegmen triangular and broad, lateral lobes slightly sclerotized and combined with second connecting membrane; internal sac membranous, with distinct dorsal, median and ventral sclerites, dorsal sclerite wedge-shaped in dorsal view, ventral sclerite short and tubular (= flagellum), median sclerite small (Fig. 52C, D).

***Female reproductive organs*** (Fig. 59A–C). Tergites 8 and 9 and sternites 8 and 9 sclerotized, posterior areas of tergite 8, sternite 8, and apodemes with pubescence, spiculum gastrale Y-shaped and long; ovipositor with dense pubescence, distal part of ovipositor cylindrical, long and with a protuberance; spermatheca simple and curved.

##### Distribution

**(Fig. 68).** China (Hunan, Yunnan, Guangxi, Guangdong, Hainan).

##### Host plant and habitat.

Host plant is unknown. One collecting place, Hongkan Reservoir in Baisha, Hainan, is located in a subtropical area. The habitat is a lake area surrounded by primeval forests with high temperature and humidity.

##### Remarks.

The species is similar to *L.jianfenglingensis*, but differs by the antennomeres 5–10 nearly 1.8× as long as wide, and brownish red with a blue metallic luster (Fig. 32D); the disc of the pronotum with two or four irregular rows of fine punctures (Fig. 42A); and the dorsal sclerite of the male genitalia is strongly sclerotized (Fig. 52C, D). In *L.jianfenglingensis*, antennomeres 5–10 are almost as long as wide, and brownish red (Fig. 30D); the disc of the pronotum has two rows of fine punctures (Fig. 40B); and the ventral sclerite of male genitalia is weakly sclerotized (Fig. 54C, D).

#### ﻿ Lilioceris rondoni

Taxon classificationAnimaliaColeopteraChrysomelidae

Kimoto & Gressitt, 1979

A1E03058-5D7C-5B07-90D0-77F9F4310683

Figs 17 , 18 , 33A–D , 43A–C , 48A–D , 63A–C , 67 , 71A, B

Lilioceris rondoni Kimoto & Gressitt, 1979: 229 (Laos: Sithandone).

##### Type material examined.

***Holotype*** of *Liliocerisrondoni* (BSM, photo), Holo / Native Collector, Bishop / Laos, Ile de Khong, 17.V.1965 / Liliocerisrondoni Kimoto & Gressitt / Liliocerisrondoni n. sp. Det. Kimoto, 19, Holo / Paratype. [in original description, collector of holotype is J. A. Rondon].

##### Other material examined.

Total 137 specimens. **China: Yunnan**: 1♀, Xishuangbanna, Menghun, 1200–1400 m / 1958.V.17, Chunpei Hong coll.; 1♀, Xishuangbanna, Xiaomengyang, 850 m / 1958.IV.15, Chunpei Hong coll.; 1♀, Xishuangbanna, Menghun, 1200 m / 1958.VI.6, Chunpei Hong coll.; 2♀, Xishuangbanna, Menghun, 1200–1400 m / 1958.X.28, Shuyong Wang coll.; 1♀, Xishuangbanna, Menghun, 1200 m / 1958.V.11, Yiran Zhang coll.; 1♀, Xishuangbanna, Menghun, 1200–1400 m / 1958.V.12, Chunpei Hong coll.; 1♀, Xishuangbanna, Menga, 1050–1080 m / 1958.VIII.16, Fuji Pu coll.; 1♀, Xishuangbanna, Menga, 1050–1080 m / 1958.V.20, Fuji Pu coll.; 1♀, Xishuangbanna, Menghun, 650–750 m / 1958.VI.13, Xuwu Meng coll.; 1♀, Xishuangbanna, Menghun, 1200–1400 m / 1958.V.21, Yiran Zhang coll.; 1♀, Xishuangbanna, Xiaomengyang, 850 m, 1957.V.17, Fuji Pu coll.; 1♀, Xishuangbanna, Menghun, 1200–1400 m / 1958.VI.1, Shuyong Wang coll.; 1♀, Xishuangbanna, Jingdong, 1170 m / 1956.VII.4; 1♀, Xishuangbanna, Mengsong, 1600 m / 1958.V.21, Chunpei Hong coll.; 1♀, Xishuangbanna, Damenglong, 650 m / 1958.VIII.8, Yiran Zhang coll.; 1♀, Xishuangbanna, Xiaomengyang, 850 m, 1958.IX.1, Leyi Zheng coll.; 1♀, Xishuangbanna, Menghai, 1200–1600 m, 1957.VIII.10, Lingchao Zang coll.; 1♀, Lancang, 1200 m, 1957.VII.29, Lingchao Zang coll.; 1♀, Xishuangbanna, Mengla, 620–650 m / 1958.V.29, Facai Zhang coll.; 1♀, Xishuangbanna, Mengla, 620–650 m / 1959.VI.10, Fuji Pu coll.; 1♀, Xishuangbanna, Damenglong, 650 m / 1958.V.5, Yiran Zhang coll.; 2♀, Xishuangbanna, Mengla, 1000 m / 1958.V.23, Fuji Pu coll.; 1♀, Xishuangbanna, Menga, 1050–1080 m / 1958.V.23, Shuyong Wang coll.; 1♀, Xishuangbanna, Menga, 1050–1080 m / 1958.VIII.12, Fuji Pu coll.; 1♀, Xishuangbanna, Menga, 1050–1080 m / 1958.VIII.22, Shuyong Wang coll.; 1♀, Xishuangbanna, Mengla, 620–650 m / 1959.V.16, Fuji Pu coll.; 1♀, Xishuangbanna, Menga, 1050–1080 m / 1958.VIII.19, Fuji Pu coll.; 1♀, Xishuangbanna, Menga, 1050–1080 m / 1958.X.19, Zhizi Chen coll.; 1♀, Xishuangbanna, Menga, 1050–1080 m / 1958.VIII.12, Fuji Pu coll.; 2♀, Xishuangbanna, Mengla, 620–650 m / 1959.V.19, Facai Zhang coll.; 1♀, Xishuangbanna, Mengla, 1050–1080 m / 1959.VIII.5, Fuji Pu coll.; 1♀, Xishuangbanna, Mengla, 1050–1080 m / 1958.V.11, Shuyong Wang coll.; 1♀, Xishuangbanna, Mengzhe, 1200 m / 1958.VII.28, Shuyong Wang coll.; 1♀, Xishuangbanna, Mengla, 620–650 m / 1959.V.5, Suofu Li coll.; 1♀, Xishuangbanna, Mengla, 620–650 m / 1959.V.19, Facai Zhang coll.; 1♀, Xishuangbanna, Damenglong, 650 m / 1958.VIII.4, Leyi Zheng coll.; 1♂, Tengchong, Qushi, Longkou, Shrubs, N25.28580, E. 98.59128 / 1478 m, 2006.6.6, Liang HB and Hu P, California Academy and IOZ, Chinese Acad. Sci; 1♂, Xishuangbanna, Menghun, 600 m / 1958.VI.12, Chunpei Hong coll.; 1♂, Xishuangbanna, Jinghong, 650 m / 1958.XI.15, Fuji Pu coll.; 1♂, Xishuangbanna, Mengla, 620–650 m / 1958.XI.15, Fuji Pu coll.; 1♂, Xishuangbanna, Xiaomengyang, 850 m / 1957.VIII.10, Shuyong Wang coll.; 1♂, Xishuangbanna, Xiaomengyang, 850 m / 1957.X.13, Lingchao Zang coll.; 1♂, Xishuangbanna, Xiaomengyang, 1200–1600 m / 1957.VIII.10, Lingchao Zang coll.; 1♂, Lancang, 1200 m, 1957.VIII.6, Lingchao Zang coll.; 1♂, Lancang, 1200 m, 1957.VIII.8, Shuyong Wang coll.; 1♂, Xishuangbanna, Mengla, 1050–1080 m / 1958.VIII.16, Fuji Pu coll.; 1♂, Xishuangbanna, Mengla, 1050–1080 m / 1958.VIII.12, Shuyong Wang coll.; 1♂, Xishuangbanna, Menga, 1050–1080 m / 1958.VIII.5, Fuji Pu coll.; 1♂, Xishuangbanna, Menga, 1050–1080 m / 1958.V.23, Shuyong Wang coll.; 1♂, Xishuangbanna, Menghun, 1200–1400 m / 1958.X.28, Shuyong Wang coll.; 1♂, Xishuangbanna, Menga, 1050–1080 m / 1958.V.23; 1♂, Xishuangbanna, Mengzhe, 1200 m / 1958.VIII.29, Fuji Pu coll.; 1♂, Xishuangbanna, Mengzhe, 1200 m / 1958.VIII.28, Fuji Pu coll.; 1♂, Puer, 1400 m, 1955.IV.4; 1♂, Xishuangbanna, Mengla, 620–650 m / 1959.V.29, Suofu Li coll.; 1♂, Xishuangbanna, Menghun, 1200–1400 m / 1958.VI.1, Shuyong Wang coll.; 1♂, Xishuangbanna, Mengzhe, 620–650 m / 1959.V.29, Fuji Pu coll.; 1♂, Xishuangbanna, Menghun, 650 m / 1958.VI.13, Leyi Zheng coll.; 1♂, Xishuangbanna, Mengla, 620–650 m / 1959.V.21, Facai Zhang coll.; 1♂, Xishuangbanna, Mengla, 620–650 m / 1959.VI.8, Suofu Li coll.; 1♂, Jingdong, 1200 m, 1957.III.18; 1♂, Jingdong, 1200 m, 1957.V.2; 1♂, Xishuangbanna, Menga, 1050–1080 m / 1958.VI.23, Shuyong Wang coll.; 1♂, Xishuangbanna, Mengsong, 1600 m / 1958.IV.26, Shuyong Wang coll.; 1♂, Xishuangbanna, Xiaomengyang, 850 m / 1957.VI.23, Lingchao Zang coll.; 1♂, Xishuangbanna, Mengla, Paozhuqin, 21.81349°N, 101.38157°E, 935 m, 2020.VI.5, Hongbin Liang and Yuan Xu coll.; 25♀32♂, Xishuangbanna, Mengla, Longlin New Village, 21.52914°N, 101.49415°E, 1066 m, 2020.VI.5–6, Hongbin Liang and Yuan Xu coll.; 1♀, Eshan, Ayi Power Station, 24.09573°N, 102.54589°E, 1444 m, 2022.VII.2, Neng Zhang coll.; 1♀1♂, Xishuangbanna, Menghai, Menga, Nanlanghe village, 22.21595°N, 100.30576°E, 1020 m, 2020.VI.1, Hongbin Liang and Yuan Xu coll.; 1♂, Simao, Xiniuping, 2021.VII.17, Pingzhou Zhu coll.; **Sichuan**: 1♀, Emei Shan, Xixiangchi, 1957.VIII.17, Keren Huang coll.; 1♀, Emei Shan, Jiulaodong, 1957.VII.26, Keren Huang coll.

##### Diagnosis.

Anterior angles of pronotum protruding, pronotal disc with four or five irregular rows of fine punctures. Humeral groove of elytra distinct, punctures of elytra sparse and diminishing posteriorly, absent on apical 1/3. Femora of legs without tooth. Lateral metasternum with a long strip of pubescence.

##### Redescription.

BL = 6.3–7.2 mm, BW = 2.5–3.5 mm. ***Body*** brownish red.

***Head*** (Figs 17, 43A). Vertex strongly convex, with a shallow groove in the middle, sparse punctate and pubescent laterally; frontoclypeal area triangular, lateral disc with sparse punctures and pubescent; labrum transverse, with sparse pubescent; antennae nearly 1/2 of body length, antennomeres 1–4 nearly globular, antennomere 2 shortest, antennomeres 5–10 nearly 1.1× as long as wide, antennomere 11 slender.

***Pronotum*** (Figs 17, 43B). Anterior angles protruding, posterior angles not protruding; sides slightly constricted in the middle; disc flat; middle of disc with four or five rows of fine punctures. Scutellum triangular and pubescent.

***Elytra*** (Figs 17, 33B). Humeri protruding, humeral groove and basal impression distinct; elytra without completely punctate striae, punctures sparse and large in the base, diminishing posteriorly, absent on apical 1/3; intervals smooth; epipleura raised, with a row of fine punctures.

***Mesosternum pubescent*.** Lateral metasternum with a long and arcuate strip of pubescence, metepisternum densely pubescent (Fig. 43C).

***Abdominal sternite*** (Fig. 33A). Lateral transverse impressions small and indistinct on sternites 1–4, other areas of sternites 1–4 densely pubescent.

***Leg*** (Fig. 33C). Femora with dense pubescence on the dorsal surface, with sparse pubescence on the ventral surface, without tooth.

***Male genitalia*** (Fig. 48A–D). Ostium occupying 1/4 length of median lobe (Fig. 48A); apex hooked (Fig. 48B); tegmen Y-shaped, basal piece of tegmen triangular and broad, lateral lobes slightly sclerotized and combined with second connecting membrane; internal sac membranous, with distinct dorsal, median and ventral sclerites, posterior part of dorsal sclerite in dorsal view slightly widen, ventral sclerite short and flaky, median sclerite slightly sclerotized (Fig. 48C, D).

***Female reproductive organs*** (Fig. 63A–C). Tergites 8 and 9, sternites 8 and 9 sclerotized, posterior areas of tergite 8, sternite 8, and apodemes with pubescence, spiculum gastrale Y-shaped and long; ovipositor with dense pubescence, distal part of ovipositor cylindrical, long and with a protuberance; spermatheca simple and hooked.

##### Distribution

**(Fig. 67).** China (Sichuan, Yunnan); Laos (Warchałowski 2011); Thailand, new record from China.

##### Host plant and habitat

**(Fig. 71A, B).** The host plant of this species is *Smilaxbracteata* Presl according to our observation in Xishuangbanna. It shares the same habitat with *L.latissima* in Baihua Shan of Xishuangbanna, Yunnan.

##### Remarks.

This species is most similar to *L.semipunctata*, but differed by the strongly convex vertex (Fig. 43A); the disc of pronotum has three or four rows of fine punctures (Fig. 43B); the posterior part of the dorsal sclerite is slightly widened in dorsal view, quadrate at the apex (Fig. 48C). In *L.semipunctata*, the vertex is slightly convex (Fig. 44A); the disc of the pronotum has two rows of fine punctures (Fig. 44B); and the posterior part of the dorsal sclerite is parallel in dorsal view, rounded at apex (Fig. 50C).

This species has obvious sexual dimorphism: the middle of the abdominal sternites have a dense pubescence in the male, but the middle of these abdo­minal sternites are smooth in the female.

#### ﻿ Lilioceris rufometallica

Taxon classificationAnimaliaColeopteraChrysomelidae

(Pic, 1923)

CFEAAAAD-CB20-5762-8862-9F14FDA43D56

Figs 20 , 21 , 34A–D , 44A–C , 49A–D , 64A–C , 68 , 72A, B

Crioceris rufometallica Pic, 1923: 10 (Vietnam: Tonkin).
Lilioceris rufometallica : Kimoto and Gressitt 1961: 56.

##### Type material examined.

***Syntype*** of *Liliocerisrufometallica* (MNHN, photo), Hoa–Binh, Tonkin / Type / ♀ / Criocerisrufometallica, Pic / Museum Paris, 1958, M. Pic coll. / Type / Syntype / Syntype, Liliocerisrufometallica (Pic, 1923) / MNHN, Paris EC17305.

##### Other material examined.

Total 75 specimens. **China: Yunnan**: 1♀1♂, Xishuangbanna, Mengsong, 1600 m / 1958.IV.26, Shuyong Wang coll.; 1♀, Xishuangbanna, Menghun, 1200–1400 m / 1958.V.12, Chunpei Hong coll.; 1♀, Xishuangbanna, Mengzhe, 1200 m / 1998.VIII.29, Fuji Pu coll.; 1♀, Xishuangbanna, Xiaomengyang, 850 m, 1957.X.22 / Lingchao Zang coll.; 1♀, Xishuangbanna, Menga, 1050–1080 m / 1958.VIII.4, Shuyong Wong coll.; 1♀, Xishuangbanna, Menga, 1050–1080 m / 1958.VIII.11, Shuyong Wong coll.; 1♂, Xishuangbanna, Damenglong, 650 m, 1958.V.5, Yiran Zhang coll.; 2♂, Xishuangbanna, Menga, 1050–1080 m / 1958.VIII.16, Fuji Pu coll.; 1♂, Xishuangbanna, Menga, 1050–1080 m / 1958.VI.6, Shuyong Wong coll.; 1♂, Yongping to Baoshan, 1955.V.5, Rong Wu coll.; 2♀2♂, Xishuangbanna, Mengla, 2020.IX.21, Yong Wang coll.; 1♀, Xishuangbanna, Menghai, 2011.IV.26, Kaiqin Li and Hongbin Liang coll.; 1♂, Xishuangbanna, Menghai, Nanlanghe, 22.21595°N, 100.30576°E, 1020 m, 2020.VI.1, Hongbin Liang and Yuan Xu coll.; 3♀1♂, Xishuangbanna, Menghai, Manguo Laozhai, 21.77355°N, 100.33148°E, 1404 m, 2021.IV.8, Hongbin Liang and Yuan Xu coll.; 2♀3♂, Lufeng, Haitian village, 24.99330°N, 102.16692°E, 1914 m, 2022.VII.31, Neng Zhang coll.; 1♀, Gongshan, Dulongjiang, Maku village, 27.68979°N, 98.30513°E, 1733 m, 2020.IX.26, Neng Zhang coll.; 2♀, Yuanyang, Shangxincheng, 2022.IV.22, Neng Zhang coll.; 2♀, Lufeng, Haitian village, 24.99330°N, 102.16692°E, 1914 m, 2021.IV.16, Hongbin Liang and Neng Zhang coll.; 5♂, Kunming, Jindian, 25.087582°N, 102.777105°E, 1954 m, 2022.VII.6, Neng Zhang coll.; 3♀6♂, Kunming, Jindian, 25.087582°N, 102.777105°E, 1954 m, 2020.VI.14, Hongbin Liang and Yuan Xu coll.; 1♀, Simao, Xiniuping, 2021.VII.28, Pingzhou Zhu coll.; 1♀, Wenshan, Gulinqin, Houcao village, 2017.X.25, Beixiao Zheng coll.; **Guangxi**: 1♀, Longzhou, Daqing Shan, 360 m / 1963.IV.25, Chunguang Wang coll.; 1♀, Longzhou, Daqing Shan, 360 m / 1963.IV.20, Shuyong Wang coll.; 1♀, Napo, Nongxin, 1000 m, 1998.IV.12, Chunsheng Wu coll.; 1♀, Fangcheng, Tongzhong, 550 m, 2000.VI.5, Jian Yao coll.; 1♂, Napo, Nongxin, 1000 m, 1998.IV.12, Haisheng Zhou coll.; **Hainan**: 1♀, Jianfengling, 1982.IV.27 / Maobin Gu coll.; 1♀, Diaoluo Shan, 1000 m / 1980.IV.23, Shuyong Wang coll.; 1♀, Jianfengling, 1964.V.10, Sikong Liu coll.; 1♀1♂, Diaoluo Shan, 1965.V.1, Sikong Liu coll.; 1♀, Jianfengling, 1983.IV.19 / Maobin Gu coll.; 1♀, Ledong, Jianfengling, Near Jianfengling Peak, 18.70971°N, 108.888764°E8 / 955m, 2007.V.6, Liang HB collector, Institute of Zoology; 1♀, Baisha, Yinggeling, Nankai, Daoyin village to Zafu Village, 2009.XI.22, Meiying Lin coll.; 1♀, Ledong, Jianfengling, The Peak, 18.71023°N, 108.87669°E / 975–1406 m, 2008.XI.25, Shi HL coll., Institute of Zoology; 1♂, Wanning, 1964.IV.18, Sikong Liu coll.; 1♀, Baisha, Gaofeng village, 19.04059°N, 109.31583°E, 886 m, 2020.VII.28, Yuan Xu coll.; 1♀, Limu Shan, 2021.III.10, Yuchen Zhao coll.; **Vietnam**: 5♀, Museum Paris, Tonkin N., Reg Dha Giang, Riviere Claire, Siebens Olivier, 1916; 1♂, Museum Paris, Tonkin N., Reg Dha Giang, Riviere Claire, Siebens Olivier, 1916; 1♀, Museum Paris, Mes du H Song-Chai, Rabier 258–95; 1♀, Tonkin; 1♀1♂, Tonkin, Hoa Binh, leg. A.de Cooman; (MNHN): Riviere Claire, Haut Tonkin Madon / Criocerisrufometallica Pic.

##### Diagnosis.

Anterior angles of pronotum protruding, pronotal disc with four or five irregular rows of fine punctures. Humeral groove of elytra distinct, punctures of elytra sparse and diminishing posteriorly, absent on apical 1/3. Meso- and meta-femora without tooth. Lateral metasternum with a long strip of pubescence.

##### Redescription.

BL = 7.0–8.5 mm, BW = 3.0–3.8 mm. ***Body*** brownish red.

**Figures 19–22.**
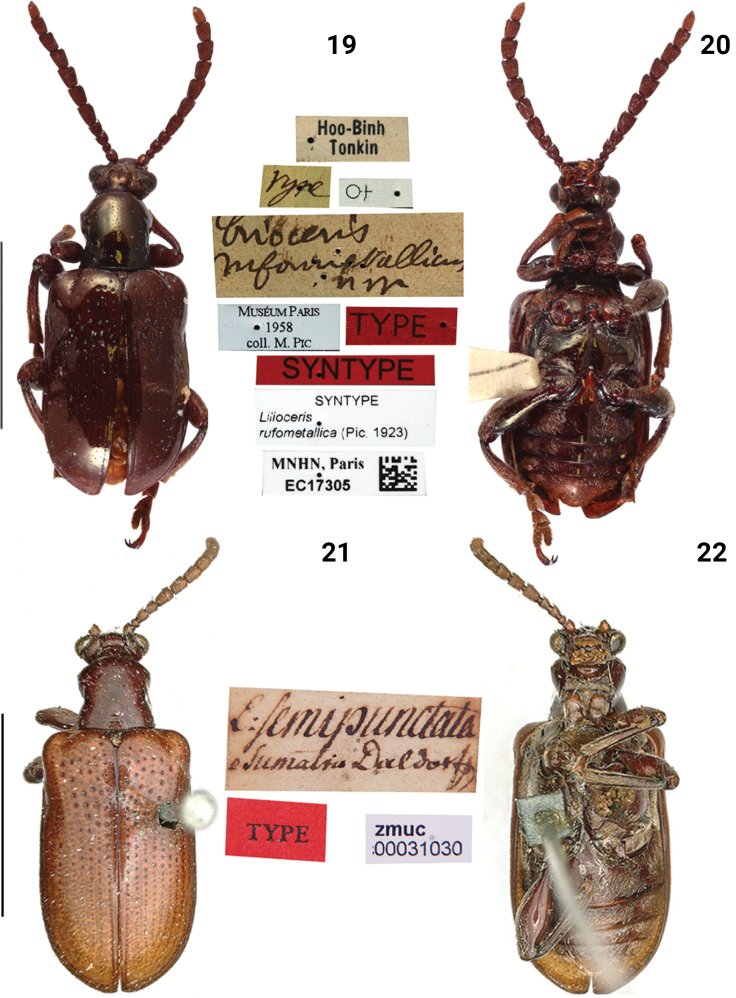
Habitus of *Lilioceris* spp. **19, 20***L.rufometallica*, syntype, Vietnam (Tonkin), photographed by Christophe Rivier (MNHN) **21, 22***L.semipunctata*, lectotype, Sumatra, photographed by Michael Kuhlmann (ZMUK). Scale bars: 5.0 mm.

***Head*** (Figs 19, 44A). Vertex flatted, with a deep groove in the middle, sparse punctate and pubescent laterally; frontoclypeal area triangular, lateral disc with sparse punctures and pubescent; labrum transverse, with sparse pubescent; antennae nearly 1/2 of body length, antennomeres 1–4 nearly globular, antennomere 2 shortest, antennomeres 5–10 almost as long as wide, antennomere 11 slender.

***Pronotum*** (Figs 19, 44B). Anterior angles protruding, posterior angles not protruding; sides slightly constricted in the middle; disc flatted; middle of disc with 3–5 rows of fine punctures. Scutellum triangular and pubescent.

***Elytra*** (Figs 19, 34B). Humeri protruding, humeral groove and basal impression distinct; punctures dense and large in the base, diminishing posteriorly, absent on apical 1/4; intervals without punctures; epipleura raised, with a sparse row of fine punctures.

***Mesosternum pubescent*.** Lateral metasternum with a long and arcuate strip of pubescence, metepisternum densely pubescent. Metepisternum densely pubescent (Fig. 44C).

***Abdominal sternite*** (Fig. 34A). Lateral transverse impressions big and distinct on sternites 1–4, other areas of sternites 1–4 densely pubescent.

***Leg*** (Fig. 34C). Femora with dense pubescence on the dorsal surface, with sparse pubescence on the ventral surface, without tooth.

***Male genitalia*** (Fig. 49A–D). Ostium occupying 1/4 length of median lobe (Fig. 49A); apex hooked (Fig. 49B); tegmen Y-shaped, basal piece of tegmen triangular and broad, lateral lobes slightly sclerotized and combined with second connecting membrane; internal sac membranous, with distinct dorsal, median and ventral sclerites, posterior part of dorsal sclerite strongly widen in dorsal view, ventral sclerite extended and curly, median sclerite small (Fig. 49C, D).

***Female reproductive organs*** (Fig. 64A–C). Tergites 8 and 9, sternites 8 and 9 sclerotized, posterior areas of tergite 8, sternite 8, and apodemes with pubescence, spiculum gastrale Y-shaped and long; ovipositor with dense pubescence, distal part of ovipositor cylindrical, long and with a protuberance; spermatheca simple and hooked.

##### Distribution

**(Fig. 68).** China (Sichuan, Yunnan, Guangxi, Hainan); Vietnam.

##### Host plant and habitat

**(Fig. 72A, B).** The host plant of this species is *Smilaxperfoliata* Lour. according to our observation in Yunnan. It lives at altitudes of 500–2000 m. One locality of this species in Menghai (Yunnan, Xishuangbanna) is situated at the subtropics. The habitat is tea plantation, adjacent to primeval forests with high temperature and humidity, and plenty of sunlight.

##### Remarks.

This species is special in this group due to its strong cupreous metallic luster, the pronotal disc having 3–5 rows of fine punctures, and lateral metasternum with a long and arcuate strip of pubescence.

#### ﻿ Lilioceris semipunctata

Taxon classificationAnimaliaColeopteraChrysomelidae

(Fabricius, 1801)

96E405E9-2055-5B33-9D8C-73AD64410A82

Figs 21 , 22 , 35A–D , 45A–C , 50A–D , 62A–C , 67 , 73A, B

Lema semipunctata Fabricius, 1801: 472 (Sumatra).
Lilioceris semipunctata : Kimoto and Gressitt 1961: 58.
Lema dehaanii Guerin–Meneville, 1844: 261 (Java). Synonymized by Kimoto and Gressitt 1979: 231.
Crioceris rufimembris Pic, 1921: 2 (China: Yunnan, syntype, male), syn. nov.

##### Type material examined.

***Lectotype*** (here selected) of *Liliocerissemipunctata* (ZMUC, photo), L.semipunctata, Sumatra, Daldorff / TYPE / zmuc 00031030 [other two syntypes: zmuc 00031032 and zmuc 00031031 are identical to *Liliocerislatissima*]. ***Syntype*** of *Liliocerisrufimembris* (MNHN, photo): Yunnan / rufimembris, Pic / type / Museum Paris, Coll. M. Pic / Type / Syntype / Syntype, Liliocerisrufimembris (Pic, 1921) / MNHN, Paris, EC15769.

##### Other material examined.

Total 451 specimens. **China: Yunnan**: 2♀, Xiaomengyang, 1000 m / 1957.V.6, Dahua Liu coll.; 1♀, Xishuangbanna, Mengla, 620–650 m / 1959.VII.9, Yiran Zhang coll.; 1♀, Yiwu toMenglun, 650 m / 1964.IV.14, Baolin Zhang coll.; 1♀, Xishuangbanna, Menghai 1200–1600 m / 1958.VII.25, Shuyong Wang coll.; 1♀, Xishuangbanna, Jinghong, 900–710 m / 1958.IV.29, Yiran Zhang coll.; 1♀, Xishuangbanna, Damenglong, 650 m / 1958.VII.11; 1♀, Xishuangbanna, Menga, 1050–1080 m / 1958.VIII.19, Fuji Pu coll.; 2♀, Xishuangbanna, 750 m / 1958.V.31, Chunpei Hong coll., Liliocerissemipunctata, det. Peiyu Yu; 2♀, Xishuangbanna, Menga, 1050–1080 m / 1958.IV.30, Shuyong Wang coll.; 1♀, Xishuangbanna, Menga, 1050–1080 m / 1958.IV.30, Fuji Pu coll.; 1♀, Xishuangbanna, Xiaomengyang, 850 m / 1958.VIII.31; 1♀, Xishuangbanna, Mengla, 620–650 m / 1959.V.8, Fuji Pu coll.; 1♀, Xishuangbanna, Damenglong, 650 m; 1♀, Xishuangbanna, Menga, 1050–1080 m / 1958.VIII.10, Shuyong Wang coll.; 1♀, Xishuangbanna, Damenglong, 650 m; 1♀, Xishuangbanna, Menga, 1050–1080 m / 1958.V.6, Fuji Pu coll.; 1♀, Xishuangbanna, Mengzhe, 1890 m / 1958.VII.7, Fuji Pu coll.; 1♀, Xiaomengyang, 850 m / 1957.VI.21, Shuyong Wang coll.; 1♀, Xishuangbanna, Mengla, 620–650 m / 1959.VII.9, Yiran Zhang coll.; 1♀, Xishuangbanna, Xiaomengyang, 850 m / 1958.VII.20, Yiran Zhang coll.; 1♀, Xishuangbanna, Mengla, 620–650 m / 1959.VII.10, Yiran Zhang coll.; 1♀, Xishuangbanna, Mengla, 620–650 m / 1959.VI.6, Fuji Pu coll.; 1♀, Xishuangbanna, Mengla, 620–650 m / 1959.V.19, Facai Zhang coll.; 1♀, Xishuangbanna, Mengzhe, 620–650 m / 1959.V.8, Fuji Pu coll.; 1♀, Xishuangbanna, Mengzhe, 850 m / 1958.VII.3, Fuji Pu coll.; 1♀, Xishuangbanna, Mengzhe, 850 m / 1958.VII.4, Shuyong Wang coll.; 2♀, Xishuangbanna, Mengzhe, 1200 m / 1958.VI.14, Shuyong Wang coll.; 1♀, Xishuangbanna, Mengzhe, 875 m / 1958.VII.4, Zhizi Chen coll.; 1♀, Xishuangbanna, Mengla, 620–650 m / 1959.V.16, Facai Zhang coll.; 1♀, Xishuangbanna, Xiaomengyang, 850 m / 1957.VI.9, Shuyong Wang coll.; 1♀, Xishuangbanna, Xiaomengyang, 850 m / 1957.VI.25, Shuyong Wang coll.; 2♀, Xishuangbanna, Mengzhe, 870 m / 1958.IX.3, Shuyong Wang coll.; 1♀, Xishuangbanna, Mengzhe, 1200 m / 1958.VI.14, Shuyong Wang coll.; 1♀, Xishuangbanna, Mengzhe, 875 m / 1958.VII.1, Fuji Pu coll.; 1♀, Xishuangbanna, Mengla, 620–650 m / 1959.V.12, Facai Zhang coll.; 1♀, Xishuangbanna, Mengla, 620–650 m / 1959.V.22, Fuji Pu coll.; 1♀, Xishuangbanna, Mengla, 620–650 m / 1959.VI.29, Facai Zhang coll.; 1♀, Xishuangbanna, Mengsong, 1600 m / 1958.VII.25, Leyi Zheng coll.; 1♀, Xishuangbanna, Mengsong, 1600 m / 1958.IX.24, Leyi Zheng coll.; 1♀, Xishuangbanna, Mengzhe, 870 m / 1958.VII.11, Fuji Pu coll.; 1♀, Menghai, 1060 m, 1980.5.1, Zuyun Guo coll.; 1♀, Xishuangbanna, Menghun, 750 m, 1958.5.30; 1♀, Xishuangbanna, Menghun, 750–950 m, 1958.VI.3; 1♀, Xishuangbanna, Menga, 800 m / 1958.VI.1, Shuyong Wang coll.; 1♀, Xishuangbanna, Xiaomengyang, 850 m / 1957.IX.3, Shuyong Wang coll.; 1♀, Xishuangbanna, Damenglong, 650 m, 1958.IV.21, Xuwu Meng; 1♀, Ruili, Nongdao, Wudian, 23.98207°N, 97.61129°E / 2012.XI.4, 990 m, Xinlei Huang leg. Inst. of Zoology, CAS.; 1♀, Nabanhe Reserve, 2008.VIII.6, 727 m / 22.16713°N, 100.65887°E, Jingxin Liu coll.; 1♀, Fugong, Shangpa Town, 26.86203°N, 98.87142°E / 1177 m, 2005.8.22, Liang HB and Zhang JF coll.; 1♀, Xishuangbanna, Mengla, 620–650 m / 1959.V.12, Facai Zhang coll.; 1♀, Xishuangbanna, Mengla, 1050–1080 m / 1958.VIII.15, Fuji Pu coll.; 1♀, Xishuangbanna, Mengla, 1050–1080 m / 1958.VIII.20, Fuji Pu coll.; 1♀, Xishuangbanna, Mengla, 1050–1080 m / 1958.VIII.16, Shuyong Wang coll.; 1♀, Xishuangbanna, Xiaomengyang, 850 m / 1957.VI.18, Shuyong Wang coll.; 1♀, Xishuangbanna, Xiaomengyang, 850 m / 1957.VII.17, Shuyong Wang coll.; 1♀, Xishuangbanna, Xiaomengyang, 850 m / 1957.VII.12, Shuyong Wang coll.; 1♀, Xishuangbanna, Xiaomengyang, 850 m / 1957.VII.9, Shuyong Wang coll.; 1♀, Xishuangbanna, Xiaomengyang, 850 m / 1957.VI.27, Lingchao Zang coll.; 1♀, Xishuangbanna, Menglun, 650 m / 1964.IV.14, Baolin Zhang coll.; 1♀, Xishuangbanna, Ganlanba, 540 m, 1957.IV.17, Fuji Pu coll.; 1♀, Baoshan to Yongping, 1955.V.28, Tianrong Huang coll.; 1♀, Wenshan / 1958.VIII.2; 1♀, Xishuangbanna, Menghun, 1200–1400 m, 1958.X.28, Shuyong Wang coll.; 2♀, Xishuangbanna, Menghun, 750 m, 1958.VI.1, Shuyong Wang coll.; 3♀, Xishuangbanna, Menghun, 750 m / 1958.VI.5, Chunpei Hong coll.; 1♀, Xishuangbanna, Menga, 800 m / 1958.IX.3, Shuyong Wang coll.; 1♀, Xishuangbanna, Menga, 1050–1080 m / 1958.VIII.16, Shuyong Wang coll.; 1♀, Xishuangbanna, Mengla, 620–650 m / 1958.VII.9, Fuji Pu coll.; 1♀, Xishuangbanna, Mengla, 620–650 m / 1958.V.30, Facai Zhang coll.; 1♀, Xishuangbanna, Damenglong, 650 m / 1958.IV.7; 1♀, Xishuangbanna, Mengla, 620–650 m / 1959.V.31, Suofu Li coll.; 1♀, Xishuangbanna, Mengla, 620–650 m / 1959.V.8, Fuji Pu coll. / host plant: Smilax sp.; 1♀, Ruili, 1979.IX.2, Mengqiao, Leyi Zheng coll. / 1400 m / Liliocerissemipunctata, det. Liang H.B.; 1♀, Tengchong, Jietou, Wujia Jidi, 1929.IV.2, 1530 m, Xiaohong Ou coll.; 1♀, Xishuangbanna, Damenglong, 650 m / 1958.VI.9, Shuyong Wang coll.; 1♀, Xishuangbanna, Mengsong, 1600 m / 1958.IV.23, Yiran Zhang coll.; 1♀, Xishuangbanna, Mengsong, 1600 m / 1958.IV.28, Fuji Pu coll.; 1♀, Xishuangbanna, Kongming Shan, 2600 m / 1957.IX.23, Lingchao Zang coll.; 2♀, Lancang, 1000 m / 1957.VII.26, Shuyong Wang coll.; 1♀, Lancang, 1100 m / 1957.VIII.8, Lingchao Zang coll.; 1♀, Xishuangbanna, Menga, 1050–1080 m / 1958.VI.1, Fuji Pu coll.; 1♀, Lancang, 1100 m / 1957.VIII.2, Lingchao Zang coll.; 1♀, Shuangjiang, 1953.VI; 1♀, 22 km northeast of Jingdong, 1957.V.9, Menchalsky [in Chinese]coll.; 1♀, Xishuangbanna, Menghun, 650 m / 1958.VI.15, Xuwu Meng coll.; 1♀, Xishuangbanna, Menga, 1000 m / 1958.V.16, Fuji Pu coll.; 1♀, Xishuangbanna, Mengla, 620–650 m / 1959.V.23, Fuji Pu coll.; 1♀, Tengchong, Qingshui, Rehai, on vegetation, 24.94861°N, 98.45181°E / 1470 m, 2006.6.1, Liang HB and Hu P coll., California Academy and IOZ, Chinese Acad. Sci.; 1♂, Xishuangbanna, Mengzhe, 870 m / 1958.IX.2, Shuyong Wang coll.; 1♂, Xishuangbanna, Mengzhe, 870 m / 1958.IX.2, Fuji Pu coll.; 1♂, Ruili, Ruili Botanical garden, 24.07230°N, 97.81944°E / 2012.X.25, 1152 m, Xinlei Huang coll.; 1♂, Ruili, Nongdao, Wudian, 23.98207°N, 97.61129°E / 2012.XI, 990 m, Xinlei Huang coll. / Liliocerislatissima, det. Liang H.B. 2022; 1♂, Xishuangbanna, Menga, 1050–1080 m / 1958.VIII.16, Shuyong Wang coll.; 1♂, Xishuangbanna, Menga, 1050–1080 m / 1958.VIII.10, Shuyong Wang coll.; 1♂, Xishuangbanna, Menga, 1050–1080 m / 1958.VIII.16, Fuji Pu coll.; 1♂, Xishuangbanna, Menghun, 1200 m / 1958.VI.28, Shuyong Wang coll.; 1♂, Xishuangbanna, Mengzhe, 870 m / 1958.IX.2, Fuji Pu coll.; 1♂, Xishuangbanna, Mengzhe, 870 m / 1958.IX.9, Fuji Pu coll.; 1♂, Xishuangbanna, Mengzhe, 870 m / 1958.VII.4, Shuyong Wang coll.; 1♂, Xishuangbanna, Mengzhe, 1200 m / 1958.IX.14, Shuyong Wang coll.; 1♂, Xiaomengyang, 850 m; 1♂, Xishuangbanna, Menghun, 1200 m / 1958.V.23, Xuwu Meng coll.; 3♂, Xishuangbanna, Mengzhe, 1200 m / 1958.VI.14, Shuyong Wang coll.; 2♂, Xishuangbanna, Mengla, 620–650 m / 1959.VII.9, Fuji Pu coll.; 1♂, Xishuangbanna, Mengla, 620–650 m / 1959.V.23, Suofu Li coll.; 1♂, Xishuangbanna, Mengsong, 1600 m / 1958.IV.24, Fuji Pu coll.; 1♂, Xishuangbanna, Menga, 800 m / 1958.V.30, Fuji Pu coll.; 1♂, Xishuangbanna, Mengla, 620–650 m / 1959.V.11, Fuji Pu coll.; 1♂, Xishuangbanna, Mengla, 620–650 m / 1959.V.16, Fuji Pu coll.; 1♂, Simao to Puwen, 950–1200 m / 1957.V.11, Guangji Hong and Zhiran Meng coll.; 1♂, Xishuangbanna, Mengzhe, 620–650 m / 1959.VII.9, Facai Zhang coll.; 1♂, Shuangjiang, 1953.VI; 1♂, 22 km northeast of Jingdong, 1957.V.9, Mengqiaciji coll.; 1♂, Xishuangbanna, Menghun, 1200 m / 1958.VI.28, Shuyong Wang coll.; 1♂, Xishuangbanna, Menghun, 750 m / 1958.VI.2; 1♂, Xishuangbanna, Mengla, 620–650 m / 1959.V.11, Facai Zhang coll.; 1♂, Xiaomengyang, 850 m / 1957.IX.13, Shuyong Wang coll.; 1♂, Xishuangbanna, Damenglong, 650 m / 1958.IV.7; 1♂, Xishuangbanna, Mengla, 620–650 m / 1959.V.16, Fuji Pu coll.; 1♂, Xiaomengyang, 850 m / 1957.VI.18, Shuyong Wang coll.; 1♂, Xiaomengyang, 620–650 m / 1959.V.5, Yiran Zhang coll.; 1♂, Xishuangbanna, Mengla, 620–650 m / 1959.V.7, Facai Zhang coll.; 2♂, Xiaomengyang, 850 m / 1957.VII.11–12, Lingchao Zang coll.; 1♂, Xishuangbanna, Damenglong, 650 m / 1958.IV.6, Yiran Zhang coll.; 2♂, Xiaomengyang, 850 m / 1957.V.7, Bonfirov [in Chinese] coll.; 1♂, Cheli [= Jinghong], Shihuiyao, 750 m, 1957.IV.27, Bonfirov [in Chinese] coll.; 1♂, Gaoligong Shan, 1200 m / 1958.VIII.22, Fulong Li coll.; 1♂, Xishuangbanna, Menglun, 650 m / 1964.V.10, Baolin Zhang coll.; 1♂, Xishuangbanna, Mengla, 620–650 m / 1959.VI.6, Fuji Pu coll.; 1♂, Xishuangbanna, Menga, 1050–1080 m / 1958.VIII.15, Fuji Pu coll.; 1♂, Puwen, 1957.III. 27, Mengqiaciji coll.; 1♂, Xishuangbanna, Menga, 1050–1080 m / 1958.V.23, Shuyong Wang coll.; 1♂, Xishuangbanna, Menglun, 650 m / 1959.VII.23, Fuji Pu coll.; 2♂, Menga, 1958.V.30 / 800 m; 1♂, Xishuangbanna, Mengla, 620–650 m / 1959.V.23, Fuji Pu coll.; 1♂, Xishuangbanna, Damenglong, 650 m / 1958.IV.18, Chunpei Hong coll.; 1♂, Xishuangbanna, Mengla, 1050–1080 m / 1958.V.20, Fuji Pu coll.; 1♂, Xishuangbanna, Mengla, 1050–1080 m / 1958.VIII.18, Fuji Pu coll.; 1♂, Xishuangbanna, Menglun, 650 m / 1959.VIII.30, Yiran Zhang coll.; 1♂, Xiaomengyang, 850 m / 1957.IX.12, Shuyong Wang coll.; 1♂, Mengla / 1982.IV.20, Shengqiao Jiang coll.; 1♂, Xishuangbanna, Mengla, 620–650 m / 1959.V.8, Fuji Pu coll.; 1♂, Xishuangbanna, Mengla, 620–650 m / 1959.VII.9, Yiran Zhang coll.; 2♂, Xishuangbanna, Mengla, 620–650 m / 1959.VI.6–9, Fuji Pu coll.; 1♂, Xishuangbanna, Menghun, 1200–1400 m / 1958.VI.3, Shuyong Wang coll.; 1♂, Xishuangbanna, Menghun, 1300 m / 1958.VI.7, Leyi Zheng coll.; 1♂, Xishuangbanna, Menghun, 750 m / 1958.VI.7; 1♂, Xishuangbanna, Menghun, 1200 m / 1958.V.23, Xuwu Meng coll.; 1♂, Xishuangbanna, Menghun, 750 m / 1958.VI.2, Xuwu Meng coll.; 1♂, Xishuangbanna, Menghun, 1200 m / 1958.V.22, Xuwu Meng coll.; 1♂, Xishuangbanna, Mengla, 620–650 m / 1959.V.23, Facai Zhang coll.; 1♂, Xishuangbanna, Menghun, 1200 m / 1958.VI.3; 1♂, Xishuangbanna, Menghun, 750 m / 1958.VI.1; 1♂, Xishuangbanna, Mengzhe, 870 m / 1958.VII.10, Shuyong Wang coll.; 1♂, Xishuangbanna, Menga, 1800 m / 1958.VI.2, Fuji Pu coll.; 2♂, Xishuangbanna, Mengla, 620–650 m / 1959.V.23, Yiran Zhang coll.; 1♂, Xishuangbanna, Mengla, 620–650 m / 1959.V.9, Facai Zhang coll.; 1♂, Xishuangbanna, Mengla, 620–650 m / 1959.VI.6, Fuji Pu coll.; 1♂, Xishuangbanna, Mengla, 620–650 m / 1959.V.4, Yiran Zhang coll.; 1♂, Xishuangbanna, Menglun, 650 m / 1959.VIII.25, Facai Zhang coll.; 1♂, Xishuangbanna, Mengla, 620–650 m / 1959.V.4, Facai Zhang coll.; 1♂, Xishuangbanna, Mengla, 620–650 m / 1959.V.19, Facai Zhang coll.; 1♂, Xishuangbanna, Menglun, 650 m / 1964.IV.29, Baolin Zhang coll.; 1♂, Cheli [= Jinghong], 620 m, 1957.IV.17, Lingchao Zang coll.; 1♂, Baoshan to Yongping, 1955.V.28, Tianrong Huang coll.; 1♂, Ruili, Dengga / 1992.VI.8, Decheng Yuan coll.; 1♂, Lincang, 1000 m / 1957.VII.26, Shuyong Wang coll.; 1♂, Lincang, 1100 m / 1957.VIII.8, Lingchao Zang coll.; 1♀, Xishuangbanna, Mengla, 620–650 m / 1958.V.30, Facai Zhang coll.; 1♀, Xishuangbanna, Mengla, 620–650 m / 1958.V.23, Facai Zhang coll.; 1♀, Xishuangbanna, Mengla, 620–650 m / 1958.V.22, Fuji Pu coll.; 1♀, Mengla / 1982.IV.20, Shengqiao Jiang coll.; 1♀, 1984.V.9, Menglun / Liliocerisimpressa (Fabricius); 1♀, Xishuangbanna, Mengla, 620–650 m / 1958.VI.6, Fuji Pu coll.; 1♀, Xishuangbanna, Mengzhe, 890 m / 1958.VII.3, Fuji Pu coll.; 1♀, Xishuangbanna, Mengzhe, 890 m / 1958.IX.5, Shuyong Wang coll.; 1♀, Xishuangbanna, Mengla, 620–650 m / 1959.V.23, Fuji Pu coll.; 1♀, Xishuangbanna, Mengzhe, 890 m / 1958.VII.8, Shuyong Wang coll.; 1♀, Xishuangbanna, Mengla, 620–650 m / 1959.VI.2, Fuji Pu coll. / Liliocerissemipunctata, det. Yuan Xu, 2021; 1♀, Xishuangbanna, Mengsong, 1600 m / 1958.IV.23, Yiran Zhang coll.; 1♀, Jinghong, 1979.X.30, Damenglong, Zuopei Lin coll. / Liliocerislatissima, det. Liang H.B. 2020; 1♀, Xishuangbanna, Mengla, 620–650 m / 1959.VI.5, Yiran Zhang coll.; 1♀, Xishuangbanna, Mengla, 620–650 m / 1959.V.20, Fuji Pu coll.; 1♀, Xishuangbanna, Mengla, 620–650 m / 1959.V.23, Fuji Pu coll.; 1♂, Xishuangbanna, Mengla, 620–650 m / 1959.V.23, Fuji Pu coll.; 1♂, Xishuangbanna, Xiaomengyang, 1957.VI.15, Shuyong Wang coll.; 1♂, Xishuangbanna, Mengzhe, 1200 m / 1958.VI.14, Shuyong Wang coll.; 1♂, Xishuangbanna, 1958.VII.8 / Mengzhe, Mengman, 870 m, Fuji Pu coll.; 1♂, Xishuangbanna, Mengzhe, 870 m / 1958.IX.7, Shuyong Wang coll.; 1♂, Xishuangbanna, Menga, 1050–1080 m / 1958.VIII.4, Fuji Pu coll.; 1♂, Xishuangbanna, Xiaomengyang, 850 m, 1957.VI.13, Shuyong Wang coll.; 1♀, Xishuangbanna, Mengla, 620–650 m / 1959.V.16, Fuji Pu coll.; 1♀, Lancang, 1000 m / 1957.VII.26, Lingchao Zang coll.; 1♀, Xishuangbanna, Menga, 1050–1080 m / 1958.VIII.16, Shuyong Wang coll.; 1♀, Xishuangbanna, Menga, 1050–1080 m / 1958.V.10; 1♀, Xishuangbanna, Menglun, 650 m / 1964.IV.14, Baolin Zhang coll.; 1♀, Xishuangbanna, Menga, 1050–1080 m / 1958.V.18, Fuji Pu coll.; 1♀, Xishuangbanna, Menga, 1050–1080 m / 1958.V.18, Fuji Pu coll.; 1♀, Xishuangbanna, Xiaomengyang, 1100 m, 1957.V.6, Dahua Liu coll.; 1♂, Xishuangbanna, Mengzhe, 800 m / 1958.VII.3, Fuji Pu coll.; 1♂, Xishuangbanna, Menghun, 750 m / 1958.VI.2; 1♂, Ruili, Ruili Botanical garden, 24.07230°N, 97.81944°E / 2012.X.27, 1152 m, Xinlei Huang coll.; 1♀1♂, Xishuangbanna, 2020.VI.30, Ying Yan coll.; 1♀, Xishuangbanna, Guomen Shan, 2019.V.11, Kaiqin Li coll.; 1♀2♂, Xishuangbanna, Menghai, Menghun, 2011.IV.26, Hongbin Liang and Kaiqin Li coll.; 8♀5♂, Xishuangbanna, Menghai, Mannong Xinzhai, 21.78233°N, 100.50706°E, 1582 m, 2021.IV.3–8, Hongbin Liang, Yuan Xu and Neng Zhang coll.; 1♂, Xishuangbanna, Guanlei, Nanlahe, 21.5796°N, 101.1879°E, 465 m, 2021.XI.30, Yong Wang coll.; 2♀4♂, Xishuangbanna, Mengla, Longlin New village, 2020.VI.5–6, 21.52914°N, 101.49415°E, 1066 m, Hongbin Liang and Yuan Xu coll.; 3♀3♂, Xishuangbanna, Xiaomengyang, Baihua Shan, 22.17720°N, 100.92428°E, 876 m, 2021.IV.3, Hongbin Liang and Yuan Xu coll.; 3♀5♂, Xishuangbanna, Menghai, Nanlanghe, 22.17720°N, 100.92428°E, 876 m, 2020.IV.9, Hongbin Liang and Yuan Xu coll.; 1♀1♂, Baoshan, Baihualing, N25.30499, 98.80008, 1622 m, 2020.IX.29, Yuan Xu coll.; 1♀, Baoshan, Baihualing, N25.30499, 98.80008, 1622 m, 2019.X.2, Hongbin Liang and Yuan Xu coll.; 1♀, Cangyuan, Banhong, 2011.V.3, Kaiqin Li coll.; 1♀, Eshan, Ayi Power Station, 24.09573°N, 102.54589°E, 1444 m, 2022.VII.2, Neng Zhang coll.; 1♀, Hekou, Huayudong, 22.67222°N, 103.93769°E, 97 m, 2021.IV.22, Hongbin Liang and Yuan Xu coll.; 3♀, Jianshui, Yunlongshan, 23.77582°N, 102.82258°E, 1645 m, 2021.IV.23, Hongbin Liang and Yuan Xu coll.; 1♀, Lvchun, Huanglian Shan, 2018.5.23, Kaiqin Li coll.; 3♀3♂, Xishuangbanna, Menghai, Nanlanghe, 22.17720°N, 100.92428°E, 876 m, 2020.VI.1, Hongbin Liang and Yuan Xu coll.; 16♀8♂, Xishuangbanna, Mengla, Longlin New village, 2020.III.30, 21.52914°N, 101.49415°E, 1066 m, Hongbin Liang and Yuan Xu coll.; 1♂, Xishuangbanna, Menghai, Mengsong, Sanmai, 21.99943°N, 100.61022°E, 1028 m, 2020.VI.2, Hongbin Liang and Yuan Xu coll.; 2♀, Ruili, Nongdao, 2019.X.10, Yuan Xu coll.; 1♂, Yingjiang, Nabang, 2012.IX.29, Hongbin Liang coll.; 6♀3♂, Eshan, Ayi Power Station, 24.09573°N, 102.54589°E, 1444 m, 2021.IV.23, Hongbin Liang and Neng Zhang coll.; 1♀, Zhenyuan, Enle, Najiu, 23.977826°N, 101.031797°E, 1359 m, 2022.VII.8, Yuan Xu and Neng Zhang coll.; 2♀, Zhenyuan, Sanzhangtian, 2022.V.28, Neng Zhang coll.; **Guangxi**: 1♂, Yangshuo, Baisha / 1963.VII.23, Shuyong Wang coll.; 2♀, Baishou, 1952.6.23; 1♀, Baishou, 1952.7.2; 1♀, Fangcheng, Fulong, 240 m, 1998.IV.20, Gexia Qiao coll.; 1♀, Longlin / 1984.V.21, Shufang Wang coll.; 1♀, Yangshuo, Baisha / 1963.VII.23, Shuyong Wang coll.; 1♀, Longzhou, Daqing Shan, 360 m, 1963.VII.23, Shuyong Wang coll.; 1♀, Longzhou, Daqing Shan, 360 m, 1963.IV.18, Shuyong Wang coll.; 1♀, Longzhou, Daqing Shan, 360 m, 1963.IV.27, Shuyong Wang coll.; 1♀, Longzhou, Daqing Shan, 360 m, 1963.IV.27, Shuyong Wang coll.; 1♀, Wuming, Daming Shan, 1963.V.21, Sikong Liu coll.; 1♂, Longzhou, Daqing Shan, 360 m, 1963.IV.25, Shuyong Wang coll.; 1♂, Wuming, Daming Shan, 1963.V.24, Jikun Yang coll.; 1♂, Longlin / 1980.IX.9, Lin Dong coll.; **Tibet**: 1♀, Mêdog, Bangxin, 29.48488°N, 95.43508°E, 1418 m, 2020.IX.13, 1♀, Mêdog, Baibung Tea Farm, 29.26310°N, 95.20983°E, 1047 m, 2020.IX.7, Hongbin Liang coll.; 1♀, Mêdog, Baibung Tea Farm, 29.26310°N, 95.20983°E, 1047 m, 2021.VI.16, Hongbin Liang and Neng Zhang coll.; 9♀7♂, Mêdog, Baibung, Gelin, 29.23370°N, 95.17707°E, 1408 m, 2020.VI.11–15, Hongbin Liang, Yuan Xu and Neng Zhang coll.; 1♀, Mêdog, Dexing, Wenlang, 29.36709°N, 95.34012°E, 1251 m, 2020.IX.4, Hongbin Liang coll.; 5♀5♂, Mêdog, Baibung, Gelin, 29.23370°N, 95.17707°E, 1408 m, 2022.VII.17–20, Hongbin Liang, Yuan Xu and Neng Zhang coll.; 1♀, Mêdog, Lagong Tea Farm, 29.31879°N, 95.31570°E, 1294 m, 2021.VI.8, Yuan Xu and Neng Zhang coll.; **Guizhou**: 2♀1♂, 1912; 1♀, Xishui, Linjiang, 300 m / IOZ and Guizhou Univ. Joint Expedition, 2000.VI.1, Song QZ coll.; 1♀, Libo, Maolan, 450 m, 1998.X.27, Xinke Yang coll.; 1♂, Libo, Maolan, 450 m, 1998.X.27, Xingke Yang coll.;3♂, Maolan, 1998.V, Qiongzhang Song coll. / semipunctata; 1♂, Libo, Maolan, 450 m, 1998.X.26, Wenzhu Li coll.; 1♂, Wangmo, 1982.VI; 1♂, Fanjing Shan, Huguo temple, 1350 m, 2001.VIII.03, Qiongzhang Song coll.; 1♀, Libo, 500–700 m / 1998.V.24–30 / Liliocerissemipunctata, Det. Peiyu Yu; 1♂, Libo, Xiaoqikong, 700 m, 1998.V.30, Runzhi Zhang coll.; 1♂, Libo, Yonggui, 700 m, 1998.V.29, Runzhi Zhang coll.; **Hainan**: 1♀, Baisha, Yinggeling, Nankai, Daoyin to Zafu village, 2009.XI.22, Meiying Lin coll.; 1♀, Changjiang, Bawang street, light trap, 19.11104, 109.08168 / 145 m, 2007.5.12, Hongbin Liang and Fuqiang Chen coll.; 1♀, Xinglong / 1963.III.12, Baolin Zhang coll.; 1♀, Tongzha, 340 m / 1960.VII.31, Changqing Li coll.; 1♀, Tongzha, 340 m / 1960.VII.31, Xuezhong Zhang coll.; 2♀, Tongzha, 340 m / 1960.VII.4, Suofu Li coll.; 1♀, 1934.10.27; 2♂, Bawangling, Dongliu, 19.054112, 109.19445 / 635 m, 2007.5.11, Hongbin Liang coll.; 1♂, Nada / 1954.IV.27, Keren Huang coll.; 1♂, Tongzha, 340 m / 1960.VI.29, Xuezhong Zhang coll.; 2♂, Tongzha, 340 m / 1960.VIII.4–5, Suofu Li coll.; 1♂, Tongzha, 340 m / 1960.VII.31, Suofu Li coll.; 2♂, Bawangling, 2019.IV.19, Run Zhou coll.; 1♀1♂, Baisha, Gaofeng village, 19.04059°N, 109.31583°E, 886 m, 2020.VII.28, Yuan Xu coll.; 2♀, Baisha, Fangza village, 19.07599°N, 109.52348°E, 438 m, 2020.VII.29, Yuan Xu coll.; 1♀1♂, Nanfaling, 18.87220°N, 109.29446°E, 537 m, 2020.VII.26, Yuan Xu coll.; **Fujian**: 1♀, Tongmuguan / 1979.VIII.4, Fusheng Huang coll. / Liliocerissemipunctata, det. Peiyu Yu / Liliocerisrufimembris, det. Liang H.B.; **Vietnam**: 1♀, Tonkin, Hoa Binh, leg. A. de Cooman, 1939.VII; 2♀, Tonkin, Hoa Binh, leg. A. de Cooman, 1940.VIII; 1♀, Tonkin, Hoa Binh, L. Ouport; 1♂, Tonkin, Hoa Binh, leg. A. de Cooman, 1940.VIII; 2♂, Tonkin, Hoa Binh, leg. A. de Cooman / Liliocerisrufimembris, det. Liang H.B; 2♂, Tonkin.

##### Diagnosis.

Anterior angles of pronotum protruding, pronotal disc with 2–5 irregular rows of fine punctures. Humeral groove of elytra distinct, punctures of elytra sparse and diminishing posteriorly, absent on apical 1/3. Femora of legs without tooth. Lateral metasternum with a long strip of pubescence.

##### Redescription.

BL = 7.5–9.0 mm, BW = 3.0–3.8 mm. ***Body*** brownish red.

***Head*** (Figs 21, 45A). Vertex slightly convex, with a shallow groove in the middle, sparse punctate and pubescent laterally; frontoclypeal area triangular, lateral disc with sparse punctures and pubescent; labrum transverse, with sparse pubescent; antennae nearly 1/2 of body length, antennomeres 1–4 nearly globular, antennomere 2 shortest, antennomeres 5–10 nearly 1.1× as long as wide, antennomere 11 slender.

***Pronotum*** (Figs 21, 45B). Anterior angles protruding, posterior angles not protruding; sides slightly constricted in the middle; disc slightly raised; middle of disc with 2–5 rows of fine punctures. Scutellum triangular and pubescent.

***Elytra*** (Figs 21, 35B). Humeri protruding, humeral groove and basal impression distinct; elytra without completely punctate striae, punctures sparse and large in the base, diminishing posteriorly, absent on apical 1/3; intervals smooth; epipleura raised, with a sparse row of fine punctures.

***Mesosternum pubescent*.** Lateral metasternum with a long and straight strip of pubescence, metepisternum densely pubescent (Fig. 45C).

***Abdominal sternite*** (Fig. 35A). Lateral transverse impressions small and indistinct on sternites 1–4, other areas of sternites 1–4 and all of sternite 5 densely pubescent.

***Leg*** (Fig. 35C). Femora with dense pubescence on the dorsal surface, with sparse pubescence on the ventral surface, without tooth.

***Male genitalia*** (Fig. 50A–D). Ostium occupying 1/4 length of median lobe (Fig. 50A); apex hooked (Fig. 50B); tegmen Y-shaped, basal piece of tegmen triangular and broad, lateral lobes slightly sclerotized and combined with second connecting membrane; internal sac membranous, with distinct dorsal, median and ventral sclerites, posterior part of dorsal sclerite paralleled in dorsal view, ventral sclerite short and flaky, median sclerite distinctly sclerotized (Fig. 50C, D).

***Female reproductive organs*** (Fig. 62A–C). Tergites 8 and 9, sternites 8 and 9 sclerotized, posterior areas of tergite 8, sternite 8, and apodemes with pubescence, spiculum gastrale Y-shaped and long; ovipositor with dense pubescence, distal part of ovipositor cylindrical, long and with a protuberance; spermatheca simple and hooked.

##### Distribution

**(Fig. 67).** China (Fujian, Guangxi, Guizhou, Hainan Tibet, Yunnan); Indonesia (Java, Sumatra); Nepal; India (Kimoto and Takizawa 1973; Bezděk and Schmitt 2017).

##### Host plant and habitat

**(Fig. 73A, B).** Host plant of this species is *Smilaxbracteata* Presl. and *Liliocerissemipunctata* shares the same habitat as *L.latissima* according to our observations in Baihua Shan of Xishuangbanna, Yunnan.

##### Remark.

Fabricius (1801: 472) described *Liliocerissemipunctata* based on specimens from Sumatra. We examined three syntype photographs of *L.semipunctata*. They obviously belong to two different species: the specimen with the identification label and locality is here designated as lectotype (Figs 21, 22). The other two specimens (TYPE / zmuc 00031032; TYPE / zmuc 00031031) with no label and no locality are here excluded from the type series. These two specimens with a glabrous metasternum are clearly different from the lectotype, but identical to *Liliocerislatissima*.

Lacordaire (1845: 559) redescribed *Lemasemipunctata* based the specimens from Java, and indicated that *Lemadehaanii* (Guérin-Méneville, 1844) was completely similar to *L.semipunctata*, and moved it to the genus *Crioceris*. Then Kimoto and Gressitt (1979: 230) synonymized *L.dehaanii* with *L.semipunctata*, and subsequent researchers followed this treatment.

**Figures 23–26.**
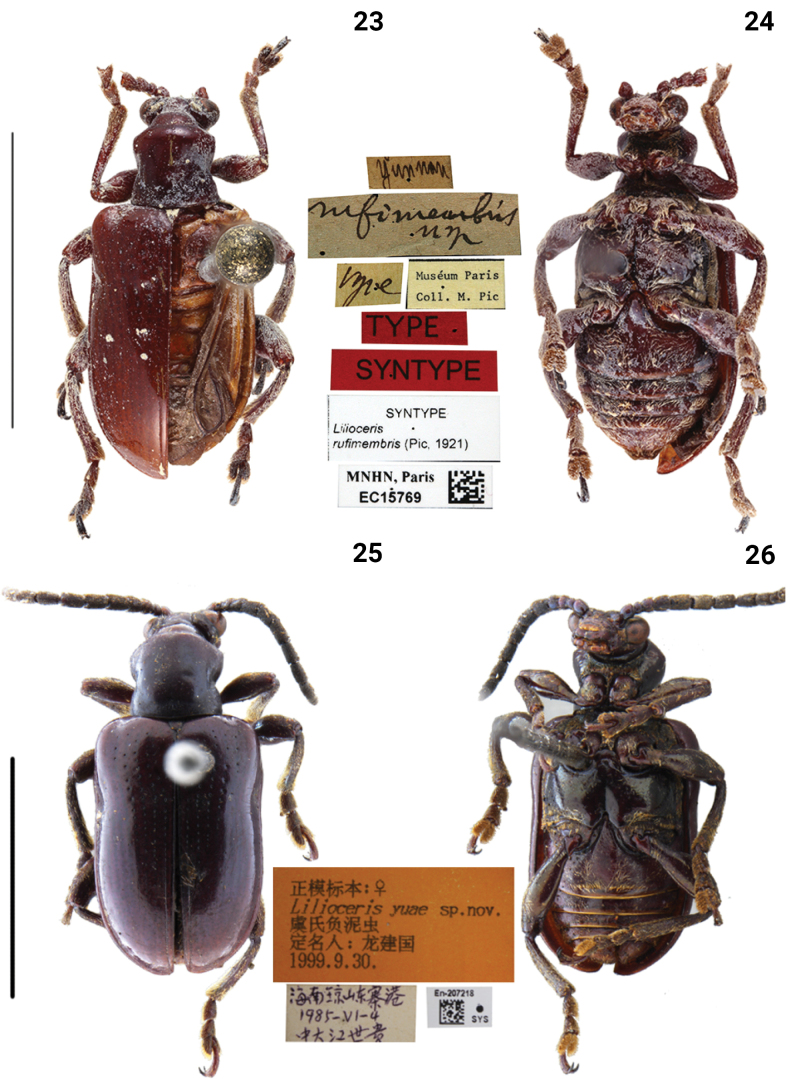
Habitus of *Lilioceris* spp. **23, 24***L.rufimembris*, syntype, China (Yunnan), photographed by Christophe Rivier (MNHN) **25, 26***L.yuae*, holotype, China (Hainan), photographed by Yuan Xu. Scale bars: 5.0 mm.

We also examined a syntype of *Liliocerisrufimembris* (Pic, 1921) in NHHN, and no significant morphological differences were found from the lectotype of *L.semipunctata*, except for two rows fine punctures on pronotum (Fig. 23), which are absent in the lectotype of *L.semipunctata* (Fig. 21). In IZCAS, there are more than 150 specimens from Yunnan that perfectly match these two types of punctures on the pronotum. We compared all these specimens and found that there were no significant morphological differences including male genitalia. Therefore, we conclude that *L.semipunctata* and *L.rufimembris* are conspecific, and that the pronotal punctures in *L.semipunctata* can be variable.

#### ﻿ Lilioceris yuae

Taxon classificationAnimaliaColeopteraChrysomelidae

Long, 2000b

728EC55F-5E3B-54C6-B41B-3FD41CFE75FF

Figs 25 , 26 , 36A–D , 46A–C , 55A–D , 66A–C , 68 , 74A–D

Lilioceris yuae Long, 2000b: 416 (China: Hainan).

##### Type material examined.

***Holotype*** of *Liliocerisyuae* (MBSU), Holotype: ♀, Liliocerisyuae sp. nov., Jianguo Long det., 1999.9.30 / Hainan, Qiong Shan, Dongzhai Gang, 1985.VI.4, Shigui Jiang coll. En–207218 SYS.

##### Other material examined.

Total 52 specimens. **China: Guangxi**: 18♀16♂, Fangchenggang, Jinhuacha Nature Reserve, 2021.III.24–29, Pingzhou Zhu coll.; 1♀, Nanning, Daming Shan, 2019.VIII.20, Meiying Lin coll.; 1♂, Fangchenggang, Shangsi, Shiwan Dashan, 2023.6.1, Yuan Xu and Neng Zhang coll.; **Guangdong**: 2♀, Dinghu Shan, 1980.VI.4–10, Yinshu Xie coll.; 2♀4♂, Zhanjiang, Dalang village, 2016.IV.26–27, Hongbin Liang coll.; **Hainan**: 1♂, Jianfengling, 1982.VIII.5 / Chengfeng Liang coll. / Liliocerisyuae, det. Liang H.B.; 1♂1♀, Qinglan / 1965.II.8, Enkong Liu coll.; 1♀, Danzhou, SC Tropical Agr. Univ., beating, 19.50664°N, 109.47883°E / 150 m, 2007.V.16, Hongbin Liang coll.; 1♀, Ledong, Jianfengling, Chahekou, 206 m, 2009.XII.4, 18.74552°N, 108.99068°E, Meiying Lin coll.; 1♀, Jianfengling, E108.8716, N18.7170, 1412 m, 2019.4.14–16, Haitian Song coll.; 2♂, Baisha, Gaofeng village, 19.04059°N, 109.31583°E, 886 m, 2020.VII.28, Yuan Xu coll.

##### Diagnosis.

Anterior angles of pronotum slightly protruding, pronotal disc with two rows of fine punctures. Humeral groove of elytra indistinct, punctures diminishing posteriorly, absent on apical 1/3. Lateral metasternum with sparse pubescence.

##### Redescription.

BL = 7.5–7.8 mm, BW = 3.5–3.8 mm. ***Body*** brownish red, head, antenna, legs and lateral metasternum with a weak blue metallic luster.

***Head*** (Figs 25, 46A). Vertex flat, with a very deep groove in the middle, sparsely punctate and pubescent laterally; frontoclypeal area triangular, lateral disc with sparse punctures and pubescent; labrum transverse, with sparse pubescent; antennae nearly 1/2 of body length, antennomeres 1–4 nearly globular, antennomere 2 shortest, antennomeres 5–10 length almost equal, flatten, antennomere 11 slender.

***Pronotum*** (Figs 25, 46B). Anterior angles protruding, posterior angles slightly protruding; sides distinctly constricted in the middle; disc flat; middle of disc with two rows of fine and shallow punctures. Scutellum triangular and pubescent.

***Elytra*** (Figs 25, 36B). Humeri protruding, humeral groove and basal impression shallow; elytra without completely punctate striae, punctures sparse, diminishing posteriorly, absent on apical 1/3; intervals smooth; epipleura raised, with a row of fine punctures.

***Mesosternum pubescent*.** Lateral metasternum with a short and oblique strip of pubescence. Metepisternum densely pubescent (Fig. 46C).

***Abdominal sternite*** (Fig. 36A). Lateral transverse impressions big and distinct on sternites 1–4, other areas of sternites 1–4 densely pubescent. Middle of first abdominal sternite with a row of distinctly long pubescence in male, and first abdominal sternite without long pubescence in female.

***Leg*** (Fig. 36C). Femora with dense pubescence on the dorsal surface, with sparse pubescence on the ventral surface, without tooth.

***Male genitalia*** (Fig. 55A–D). Ostium occupying 1/4 length of median lobe (Fig. 55A); apex slightly hooked (Fig. 55B); tegmen Y-shaped, basal piece of tegmen oval and narrow, lateral lobes slightly sclerotized and combined with second connecting membrane; internal sac membranous, with distinct dorsal and ventral sclerites, posterior part of dorsal sclerite in dorsal view parallel, ventral sclerite extended and tubular (= flagellum), median sclerite very small (Fig. 55C, D).

***Female reproductive organs*** (Fig. 66A–C). Tergites 8 and 9, sternites 8 and 9 sclerotized, posterior areas of tergite 8, sternite 8, and apodemes with pubescence, spiculum gastrale Y-shaped and long; ovipositor with dense pubescence, distal part of ovipositor cylindrical, long and with a protuberance; spermatheca simple and curved.

##### Distribution

**(Fig. 68).** China (Guangxi, Guangdong, Hainan).

##### Host plant and habitat

**(Fig. 74A–D).** This species feeds on *Smilaxlanceifolia* Roxb. according to our observation in Hainan. One locality of this species, near a road of Gaofeng village (Hainan, Baisha), is situated at the subtropics. The habitat is secondary forests, which is characterized by high temperature and humidity, plentiful precipitation, and much sunlight. The forests are open and composed of tall trees, woody vines, and many weeds.

##### Remarks.

*Liliocerisyuae* is very similar to *L.cyanicollis* but differs by the body color and pubescence on the metasternum; the body of *L.yuae* is brownish red, and only the head, antenna, leg, and lateral metasternum have a very weak blue metallic luster; lateroposterior corner of metepisternum with sparse pubescence. In *L.cyanicollis*, the elytra and abdomen are brownish red, pronotum is brownish red or blue, and the other areas are blue with a distinctly blue metallic luster; and the metepisternum is glabrous. However, the male genitalia and the female reproductive organs of these two species are very similar. We temporarily treat *L.yuae* as distinct species, as more material is needed to conclusively elucidate their relationship.

**Figures 27–31.**
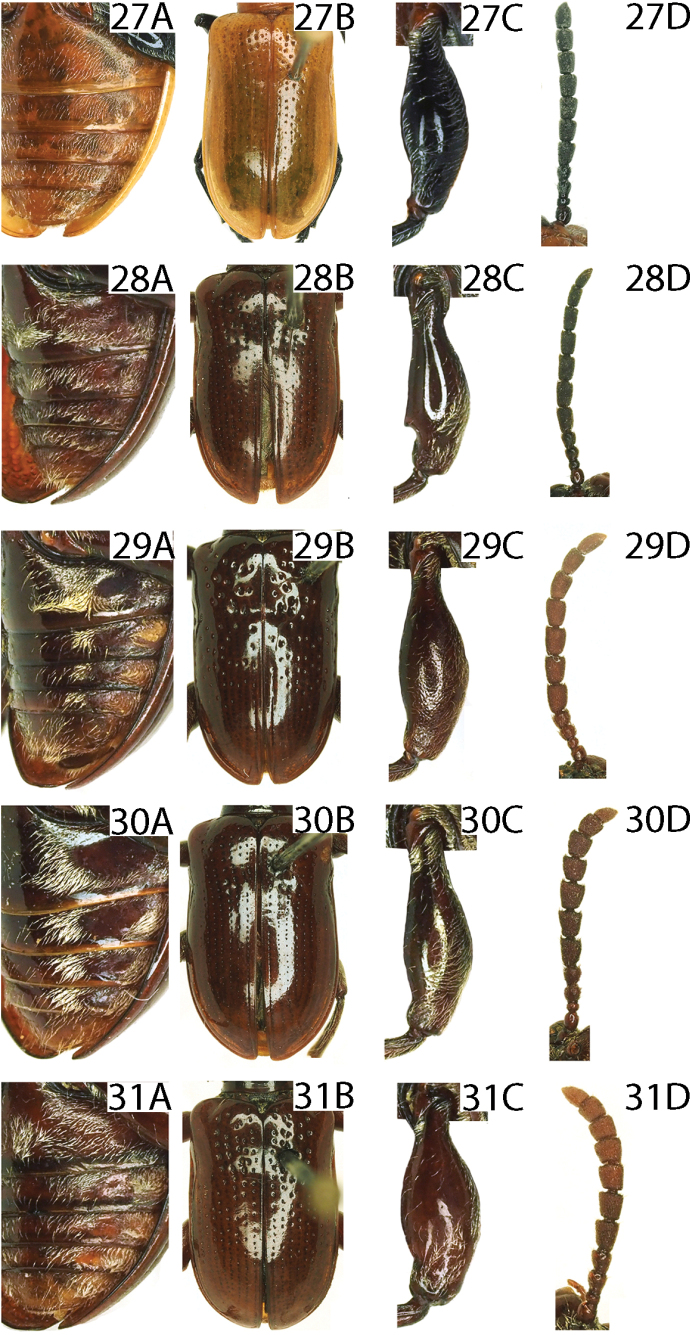
Abdominal sternites, elytra, leg, and antennae of *Lilioceris* spp. **27***L.consentanea*, ♂, China (Hainan) **28***L.dentifemoralis*, ♂, China (Hainan) **29***L.discrepens*, ♂, China (Yunnan) **30***L.jianfenglingensis*, ♂, China (Yunnan) **31***L.latissima*, ♂, China (Hainan) **A** abdominal sternites **B** elytra **C** leg **D** antennae.

**Figures 32–36.**
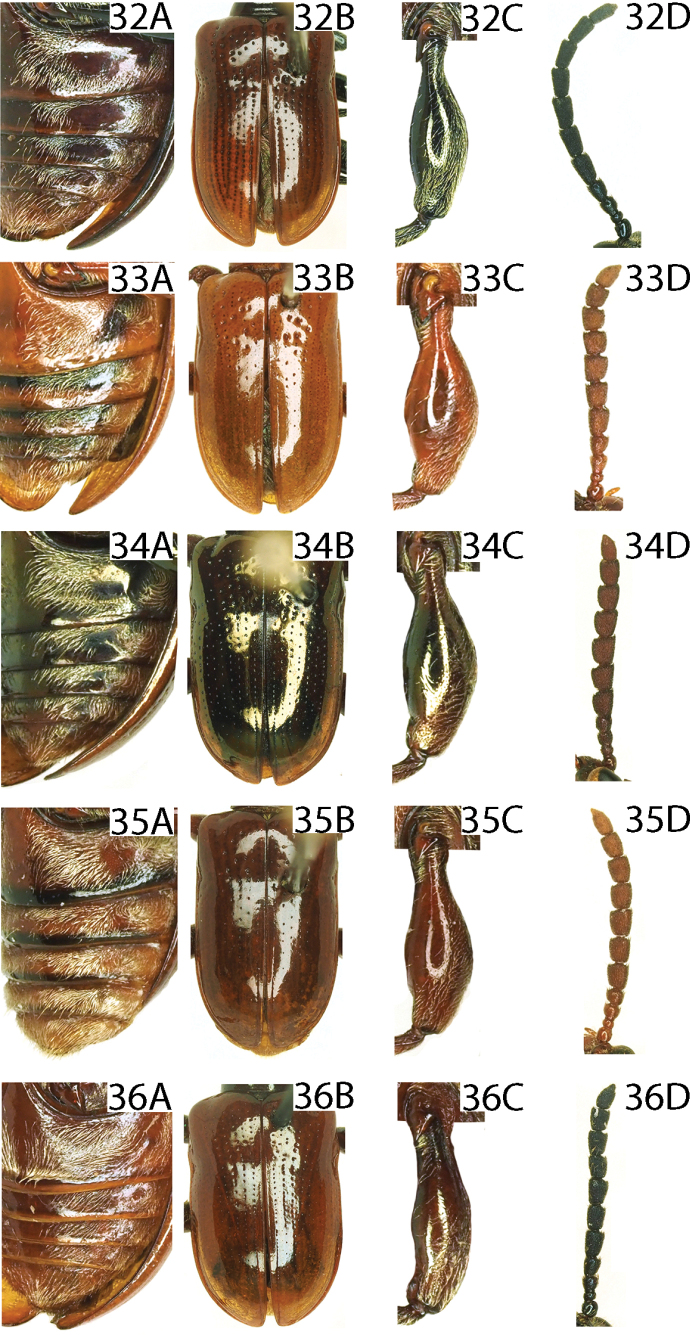
Abdominal sternites, elytra, leg, and antennae of *Lilioceris* spp. **32***L.lianzhouensis*, ♂, China (Hainan) **33***L.rondoni*, ♂, China (Yunnan) **34***L.rufometallica*, ♀, China (Hainan) **35***L.semipunctata*, ♂, China (Yunnan) **36***L.yuae*, ♀, China (Hainan) **A** abdominal sternites **B** elytra **C** leg **D** antennae.

**Figures 37–46.**
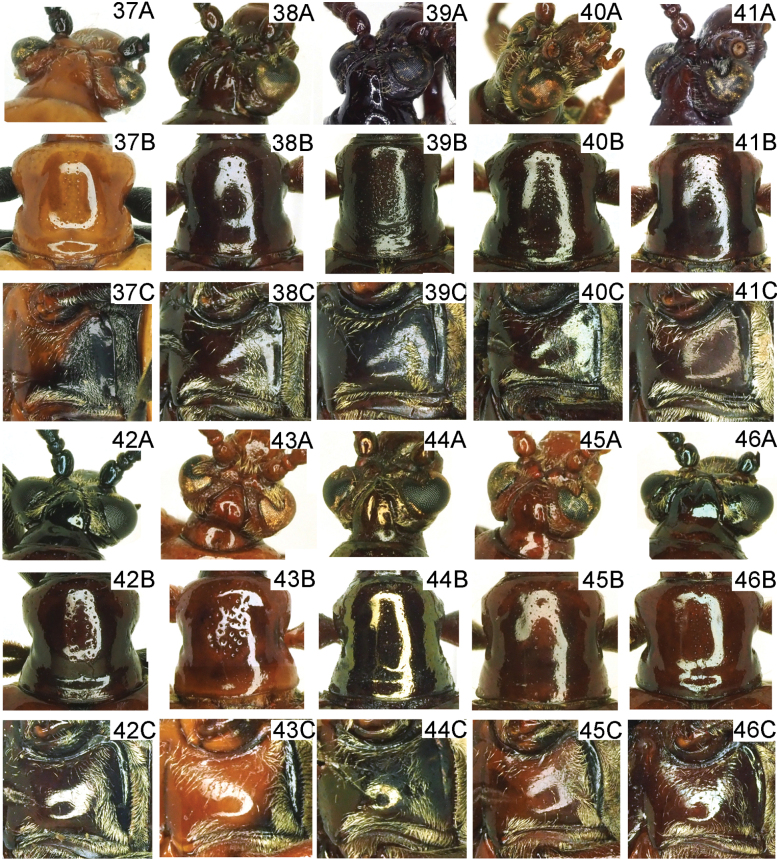
Head, pronotum, abdominal sternites of *Lilioceris* spp. **37***L.consentanea*, ♂, China (Hainan) **38***L.dentifemoralis*, ♂, China (Hainan) **39***L.discrepens*, ♂, China (Yunnan) **40***L.jianfenglingensis*, ♂, China (Hainan) **41***L.latissima*, ♀, China (Yunnan) **42***L.lianzhouensis*, ♀, China (Yunnan) **43***L.rondoni*, ♂, China (Hainan) **44***L.rufometallica*, ♀, China (Yunnan) **45***L.semipunctata*, ♀, China (Yunnan) **46***L.yuae*, ♀, China (Hainan) **A** head **B** pronotum **C** abdominal sternite.

**Figures 47–50.**
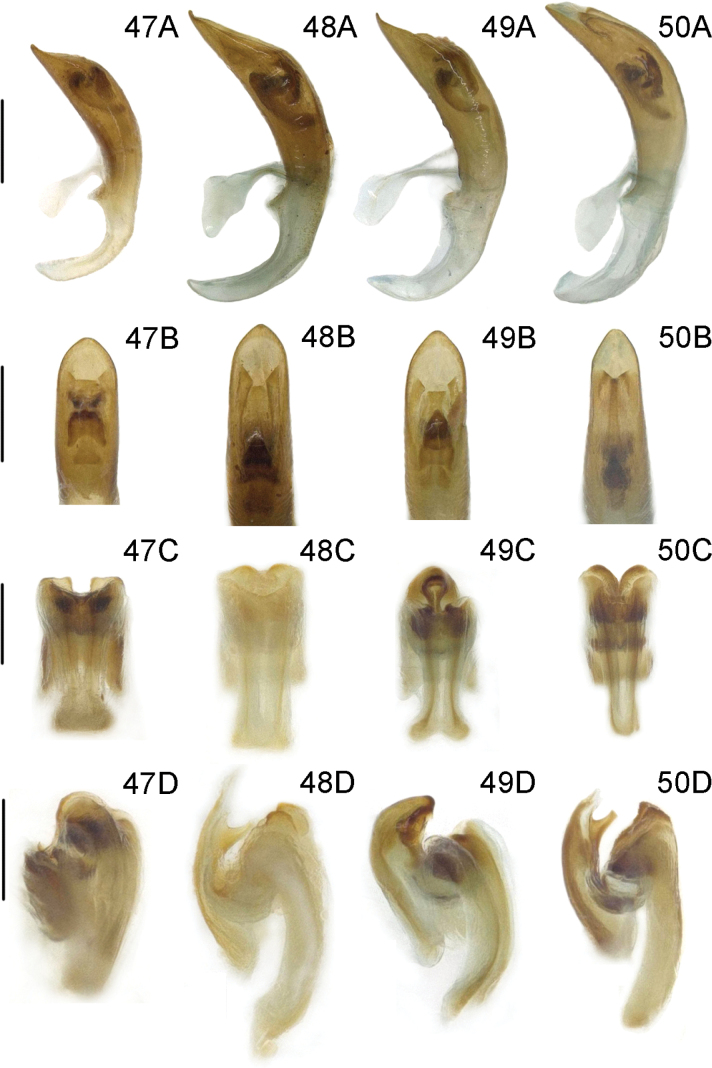
Male genitalia of *Lilioceris* spp. **47***L.dentifemoralis*, China (Hainan) **48***L.rondoni*, China (Yunnan) **49***L.rufometallica*, China (Yunnan) **50***L.semipunctata*, China (Yunnan) **A** aedeagus, lateral view **B** aedeagus, dorsal view **C** sclerites in internal sac, lateral view **D** dorsal sclerite, dorsal view. Scale bars: 0.2 mm.

**Figures 51–56.**
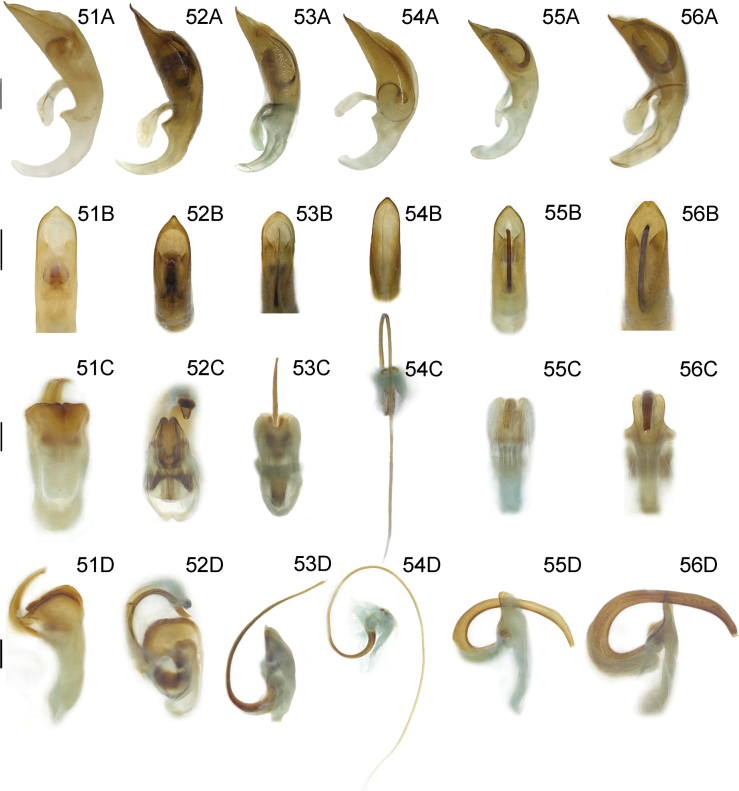
Male genitalia of *Lilioceris* spp. **51***L.discrepens*, China (Hainan) **52***L.lianzhouensis*, China (Yunnan) **53***L.latissima*, China (Yunnan) **54***L.jianfenglingensis*, China (Yunnan) **55***L.yuae*, China (Hainan) **56***L.consentanea*, China (Yunnan) **A** aedeagus, lateral view **B** aedeagus, dorsal view **C** sclerites in internal sac, lateral view **D** dorsal sclerite, dorsal view. Scale bars: 0.2 mm.

**Figures 57–66.**
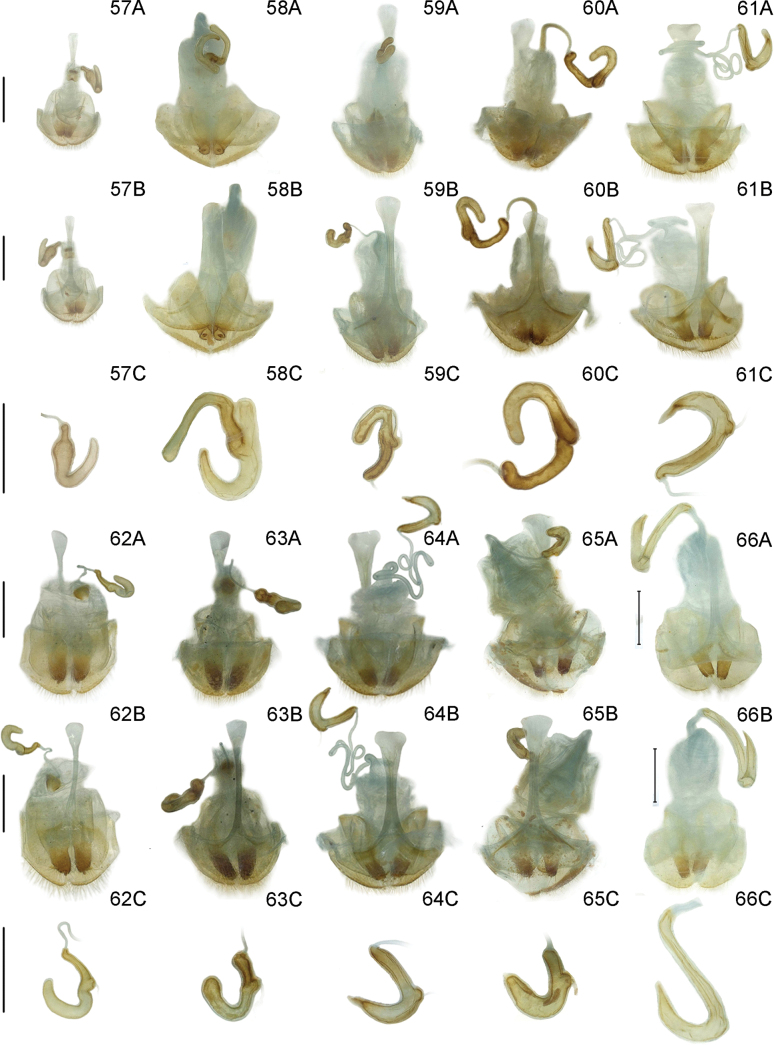
Female reproductive organs of *Lilioceris* spp. **57***L.dentifemoralis*, China (Hainan) **58***L.consentanea*, China (Hainan) **59***L.lianzhouensis*, China (Yunnan: Wuding) **60***L.latissima*, Japan (Tokyo) **61***L.jianfenglingensis*, China (Yunnan) **62***L.semipunctata*, China (Yunnan: Wuding) **63***L.rondoni*, China (Yunnan) **64***L.rufometallica*, China (Yunnan) **65***L.discrepens*, China (Yunnan) **66***L.yuae*, China (Hainan) **A** dorsal view **B** ventral view **C** spermatheca. Scale bars: 0.5 mm.

**Figure 67.**
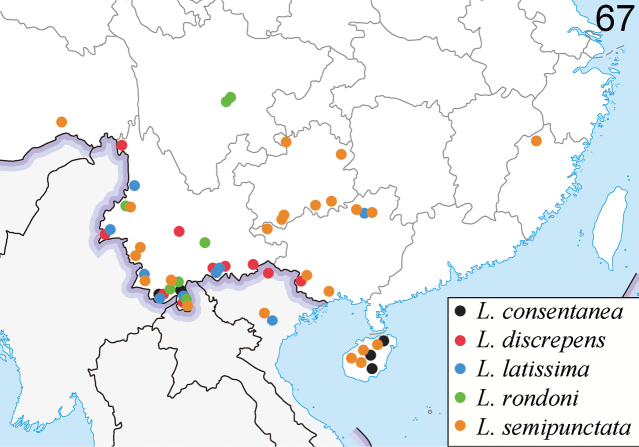
Distribution map of *Lilioceris* spp. (all marked location information is derived from the labels of available specimens).

**Figure 68.**
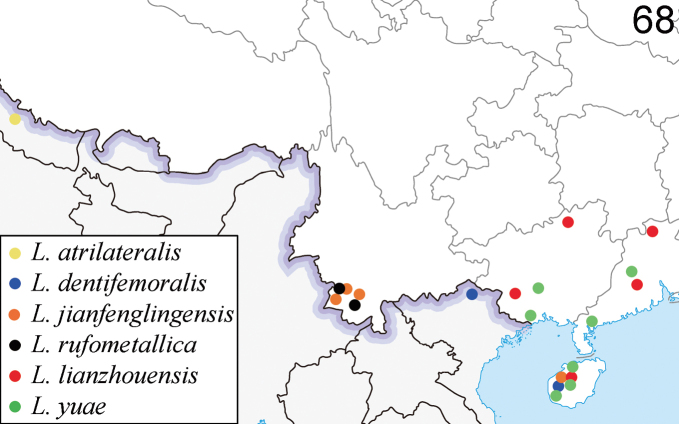
Distribution map of *Lilioceris* spp. (all marked location information is derived from the labels of available specimens).

**Figure 69.**
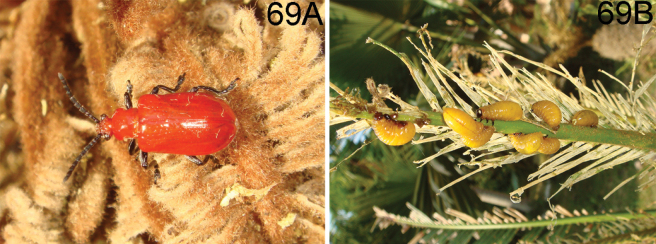
*Liliocerisconsentanea* in China (Hainan: Yinggeling), 1 April 2010, photographed by Meiying Lin **A** adult **B** larva.

**Figure 70.**
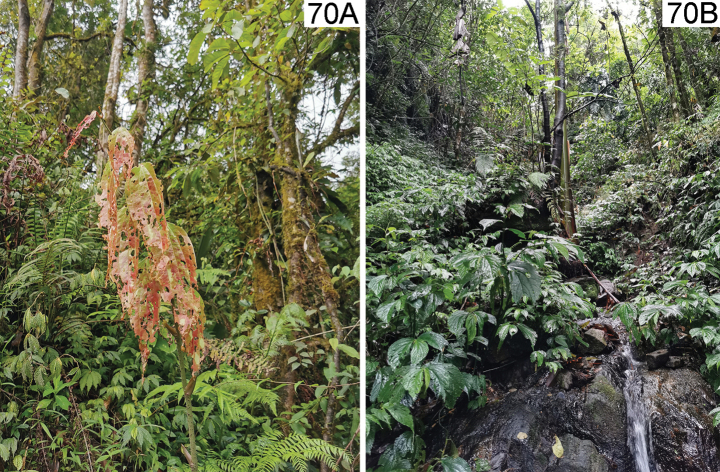
*Liliocerisdiscrepens* in China (Yunnan: Gongshan), 31 May 2021, photographed by HB Liang **A** host plant **B** habitat.

**Figure 71.**
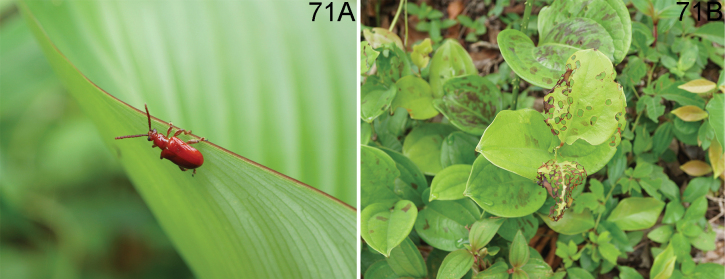
*Liliocerisrondoni* in China (Yunnan: Mengla), 2020.6.6, photographed by Y Xu **A** adult **B** host plant.

**Figure 72.**
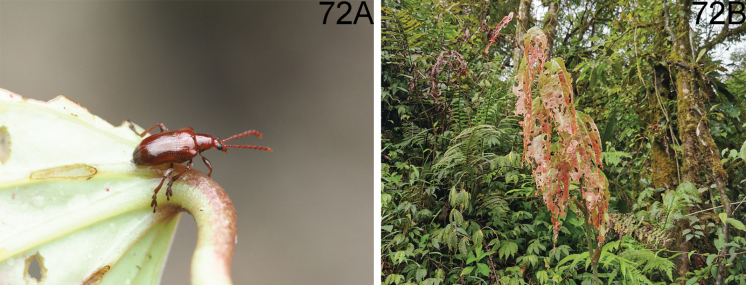
*Liliocerisrufometallica* in China (Yunnan: Gongshan), 31 May 2021, photographed by Y Xu and HB Liang **A** adult **B** host plant. (*Liliocerisrufometallica* lives together with *L.discrepens* at this site).

**Figure 73.**
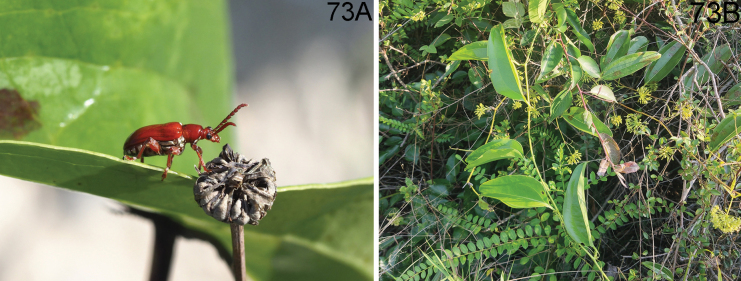
*Liliocerissemipunctata* in China (Yunnan: Jianshui), 22 April 2021, photographed by Y Xu. **A** adult **B** host plant.

**Figure 74.**
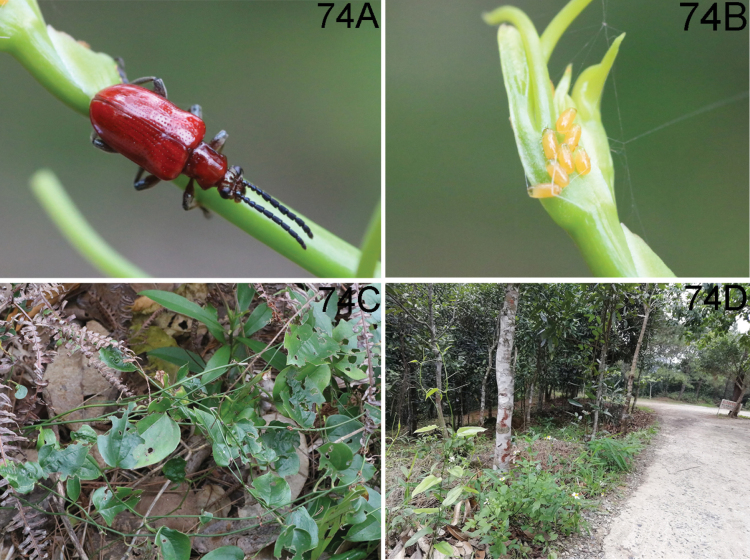
*Liliocerisyuae* in China (Guangxi: Fangchenggang), 24 March 2021, photographed by Pingzhou Zhu **A** adult **B** eggs **C** host plant **D** habitat.

## Supplementary Material

XML Treatment for Lilioceris atrilateralis

XML Treatment for Lilioceris consentanea

XML Treatment for Lilioceris dentifemoralis

XML Treatment for Lilioceris discrepens

XML Treatment for Lilioceris jianfenglingensis

XML Treatment for Lilioceris latissima

XML Treatment for Lilioceris lianzhouensis

XML Treatment for Lilioceris rondoni

XML Treatment for Lilioceris rufometallica

XML Treatment for Lilioceris semipunctata

XML Treatment for Lilioceris yuae

## ﻿Additional information

### Author ORCIDs

Yuan Xu ￼ https://orcid.org/0009-0000-3103-9125

Hongbin Liang ￼ https://orcid.org/0000-0002-9668-1167

### Data availability

All of the data that support the findings of this study are available in the main text.
